# Persistent selection on size explains micro- and macroevolutionary alignments in fly wings

**DOI:** 10.1073/pnas.2612940123

**Published:** 2026-06-18

**Authors:** Haoran Cai

**Affiliations:** ^a^ Department of Ecology and Evolutionary Biology, University of California, Los Angeles, CA 90095-1606

**Keywords:** pleiotropy, apparent selection, rate paradox, modularity, *Drosophila* wing

## Abstract

The alignment among mutational variance (*M*), standing genetic variance (*G*), and macroevolutionary divergence (*R*) in *Drosophila* wing shape poses a rate paradox under a simple constraint hypothesis: Evolution follows mutational lines of least resistance, yet proceeds orders of magnitude slower than the abundant genetic variation would permit. This is difficult to reconcile with a simple constraint view in which long-term evolution merely tracks the amount of available variation in each direction. Previous explanations invoke deleterious pleiotropy on unmeasured traits or correlational selection on trait combinations, but recent empirical work finds little evidence of fitness costs beyond flight performance. Here, by reanalyzing published data, I show that wing size shows the hallmark of the primary selection target: Among all wing traits, size exhibits the lowest ratio of standing genetic to mutational variance, indicating the strongest selective depletion. Based on this empirical observation, I develop a single-axis selection model in which natural selection targets only a single trait while all other traits evolve as correlated byproducts via within-module pleiotropy. This minimal model reproduces both the observed *M*–*G*–*R* alignment and slower-than-neutral divergence rates, explaining micro- and macroevolutionary patterns in fly wings without invoking complex adaptive landscapes.

## Significance

A fundamental question in biology is whether large-scale evolutionary patterns arise from the same processes that drive change within populations. Fly wing shape offers a striking test case: The directions of genetic variation and species divergence are closely aligned, yet wing shape evolves far more slowly than expected—a rate paradox under a simple constraint hypothesis. I show that this paradox can be explained by single-axis selection, which constrains other nonselected traits through pleiotropy. More broadly, the results suggest that multivariate constraint can emerge from selection on a single trait whenever mutations are pleiotropic within developmental modules. This implies that the alignment between micro- and macroevolution may often reflect simple, low-dimensional selection rather than complex adaptive landscapes.

A persistent question in evolutionary biology concerns the relationship between microevolution and macroevolution: Can macroevolutionary patterns be explained by microevolutionary processes, and conversely, can population-level changes be predicted from macroevolutionary trends (1–7)? Microevolution refers to the genetic and phenotypic changes within populations driven by mutation, selection, migration, and drift. Macroevolution describes the broader dynamics of life, including the origination and extinction of lineages and large-scale patterns of phenotypic divergence. While attempts to quantitatively link these two scales have yielded mixed success, a comprehensive framework explaining these variable outcomes remains elusive (8).

A notable success in aligning these scales comes from the evolution of wing shape in Drosophila and other Diptera. In this system, a strong positive correlation is consistently observed between the structure of mutational variance (the M-matrix), standing additive genetic variance (the G-matrix), and rates of macroevolutionary divergence (the R-matrix) (9). This alignment across different evolutionary timescales offers compelling evidence that nonrandom phenotypic variation generated by mutation is profoundly linked to long-term evolutionary outcomes.

This alignment, however, presents a paradox under a simple constraint view: If evolutionary trajectories simply follow lines of least resistance, they should proceed much faster given the abundant genetic variation available. Instead, observed macroevolutionary rates are orders of magnitude slower than theoretically permitted, which challenges the simple constraint-based hypothesis (9).

One proposed resolution is that most mutational variation is effectively unusable due to deleterious pleiotropic effects on unmeasured traits (9). This “pleiotropic constraint” hypothesis predicts genetic covariation between wing shape and fitness components that are functionally unrelated to wing shape or flight. However, an indirect evaluation of this hypothesis in *Sepsis punctum* found little evidence for such covariances, although the authors of that study note their power to detect subtle associations was limited (10).

Notably, the slow observed rates challenge the simple constraint explanation but pose no difficulty for selection-based accounts: If selection actively channels evolution along particular directions, slow rates are an expected consequence rather than a puzzle. A second, equally critical aspect is that the alignment between divergence and variation persists over timescales far longer than expected if the direction of selection were arbitrary with respect to *M* and *G*. Several alternative hypotheses have accordingly been proposed:

1. **Moving corridor model** (also known as “selective-lines-of-least-resistance”): Net directional selection is concentrated along a major phenotypic axis, while stabilizing selection limits departures in orthogonal directions. The multivariate fitness optimum wanders over geological time, but its trajectory is constrained to “corridors” that roughly parallel the primary axis of mutational variance (11, 12).
2. **Correlational selection**: Persistent selection on trait combinations (e.g., allometric relationships) shapes both the mutational and genetic variance–covariance matrices, so that divergence naturally aligns with the direction of covariation maintained by selection (10).
3. **Multidimensional fluctuating selection**: The multivariate optimum fluctuates rapidly across many trait dimensions (3). Because the population’s tracking response is filtered through the *G* matrix, evolutionary excursions are larger along axes of high genetic variance. Time-averaging these rapid fluctuations yields a divergence matrix (*R*) that naturally aligns with *G* and *M*. However, as explicitly noted in ref. 3, this mechanism assumes that all diverging populations remain within a single global adaptive zone, and it may therefore be insufficient to account for the apparent phylogenetic signal observed in fly wing evolution.

Here, I propose a single-axis selection model as a parsimonious alternative to these hypotheses in fly wing evolution. Like the above models, this model assumes selection is invoked, but posits that selection is concentrated primarily on a single axis. This framework asks whether complex adaptive landscape or multivariate selection is necessary to reproduce the observed patterns. Using individual-based simulations parameterized with empirical mutational variance–covariance matrices, I show that the single-axis selection model is sufficient to reproduce the observed *M*-*G*-*R* alignment and generate slower-than-neutral divergence rates. Furthermore, Model predictions together with empirical evidence suggest that wing size is the primary target of selection, effectively experiencing the strongest selection pressure among all wing traits.

## Results

### Wing Size as the Primary Selection Target Among Wing Traits.

**Fig. 1.**
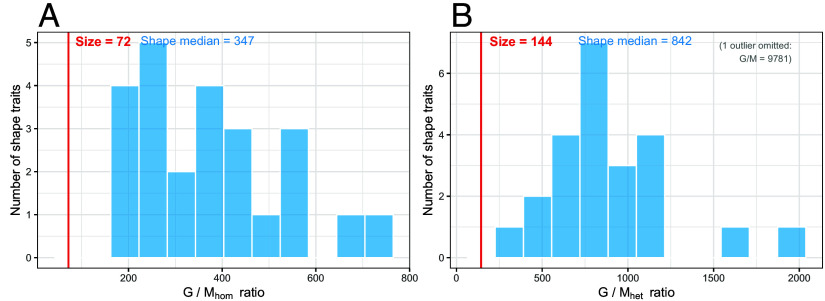
Empirical G/M ratio for size vs. shape traits in *D. melanogaster*. Distribution of per-trait $G_{\mathrm{ii}}/M_{\mathrm{ii}}$ ratios across the 24 shape traits (blue histogram) computed from the empirical *G* and *M* matrices of ref. 9. The vertical red line marks the G/M ratio of wing size. (*A*) $G/M_{\text{hom}}$ (homozygous mutational variance): size ratio =72, shape median =347; (*B*) $G/M_{\text{het}}$ (heterozygous mutational variance): size ratio =144, shape median =842. Under both estimates, size has the lowest G/M ratio of all 25 traits (rank 1/25).

Wing size is a strong candidate for the primary selection target among all wing traits within the module, given its central role in aerodynamic performance and other fitness-related contexts, as discussed below. To test this expectation, I used the empirical *G* and *M* matrices estimated for *Drosophila melanogaster* wing traits from ref. 9 and computed the per-trait ratio $G_{\mathrm{ii}}/M_{\mathrm{ii}}$, which measures how much standing genetic variance is maintained per unit of mutational input. Under pure drift, this ratio should be approximately equal for all traits; persistent selection on a trait preferentially erodes its genetic variance and depresses its G/M ratio below the neutral baseline. The G/M ratio for wing size is the lowest of all 25 traits, under both the homozygous ($G/M_{\text{hom}}=72$; shape median =347) and heterozygous ($G/M_{\text{het}}=144$; shape median =842) mutational variance estimates (Fig. 1). Size retains only *∼*21% ($M_{\text{hom}}$) to *∼*17% ($M_{\text{het}}$) as much standing genetic variance per unit of mutational input as the median shape trait. Because standing genetic variance reflects a balance between mutational input and selective removal, the uniquely low G/M ratio of size is consistent with it being the primary target of selection, effectively under the strongest selection.

### Single-Axis Selection Recapitulates the Empirical Alignment Between Divergence and Variation.

Based on this observation, here, I propose a single-axis selection model in which only one trait is under persistent selection and all others evolve as correlated byproducts of within-module pleiotropy. Given stable *M* over macroevolutionary time, the divergence matrix *R* should align with both *M* and *G*.

To test whether such a single-axis selection model can reproduce the empirical alignment patterns observed in fly wings, I conducted individual-based simulations using the empirical mutational variance–covariance matrix *M* from ref. 9. The simulation protocol was as follows: 20 replicate populations were initialized from an ancestral population experiencing a burn-in session. To allow populations to diverge, each replicate was assigned an optimum moving rate drawn from a normal distribution with mean zero and SD *σ*. This means the optimum translates at a constant velocity for a given lineage, $\theta_{i}\left(t\right)=v_{i}t$, effectively generating a sustained displacement from the optimum rather than a Brownian motion random walk. This design enables populations to track different selective targets at different speeds, generating among-lineage divergence in the primary trait (e.g., size). Larger *σ* values produce faster and more variable divergence among replicates, while smaller *σ* values constrain populations to remain closer to the ancestral optimum. The divergence matrix *R* was computed from the among-population variance in trait means, while the within-population genetic variance–covariance matrix *G* was recorded at the final generation and averaged across 20 replicate populations. Wing size was designated as the primary trait under persistent selection with a moving optimum.

To quantify the alignment between matrices, I employed common subspace analysis following previous approaches (14). This method projects the matrices onto a common set of orthogonal axes (e.g., the eigenvectors of *M*) and compares the variance along each axis on a logarithmic scale. A strong linear relationship indicates proportional scaling of variances—the signature of alignment between matrices.

**Fig. 2.**
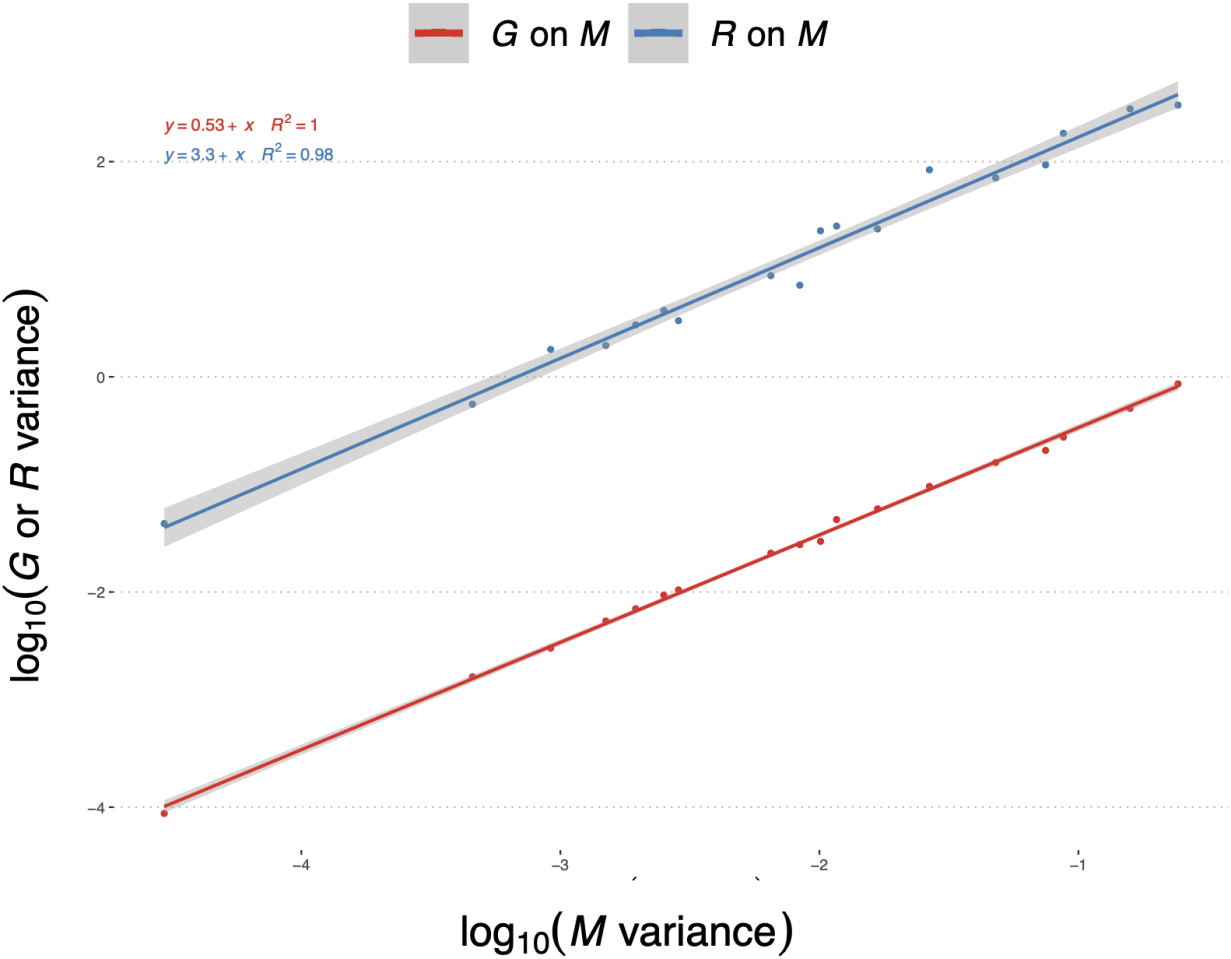
The single-axis selection model reproduces the empirical alignment between *M*, *G*, and *R*. Common subspace analysis comparing the divergence matrix *R* (blue) and genetic variance matrix *G* (red) to the mutational variance matrix *M*. Points represent $\log_{10}$ variance along the eigenvectors of *M*; lines indicate ordinary least squares regression. Strong positive relationships indicate that both *R* and *G* are aligned with the mutational architecture. Only the upper 18 eigenvectors of *M* were used; wing size was not included in the analyses. Shaded areas show 95% CI. Simulations used $V_{s}=0.05$ on wing size, σ=0.0001, N=500, and 20 replicate populations evolved for 20,000 generations.

The results show that the single-axis model selecting on size successfully reproduces the empirical patterns (Fig. 2). When comparing $\log_{10}$-transformed variances along the principal axes of *M*, both *R* and *G* showed strong positive correlations with *M*. This alignment was robust across two orders of magnitude variation in selection strength on the primary trait (*SI Appendix*, Fig. S5) and across different reference matrices (*SI Appendix*, Fig. S6; See ref. 15). Notably, this model-predicted alignment does not rely on selection targeting wing size; it is also robust when selection targets a wing-shape trait (*SI Appendix*, section C and Fig. S7). The above alignment analyses exclude wing size from the matrices, so that the common subspace comparisons specifically test whether selection on size induces proportional scaling of the multivariate covariance structure among the 24 indirectly selected shape dimensions (including size in the matrices retains similarly strong alignment). These findings indicate that a model of univariate selection, combined with stable mutational covariances, is sufficient to generate the observed *M*-*G*-*R* alignment in fly wing without invoking complex multivariate selection or correlational selection on shape.

### Depletion of Genetic Variance in the Selection Target.

To confirm that the targeted depletion of genetic variance in size from empirical observation (Fig. 1) is a direct expectation of the single-axis selection model, I examined the ratio $G_{\mathrm{ii}}/M_{\mathrm{ii}}$ for each trait *i* in the simulated populations. This ratio reflects the amount of standing genetic variance maintained per unit of mutational input.

**Fig. 3.**
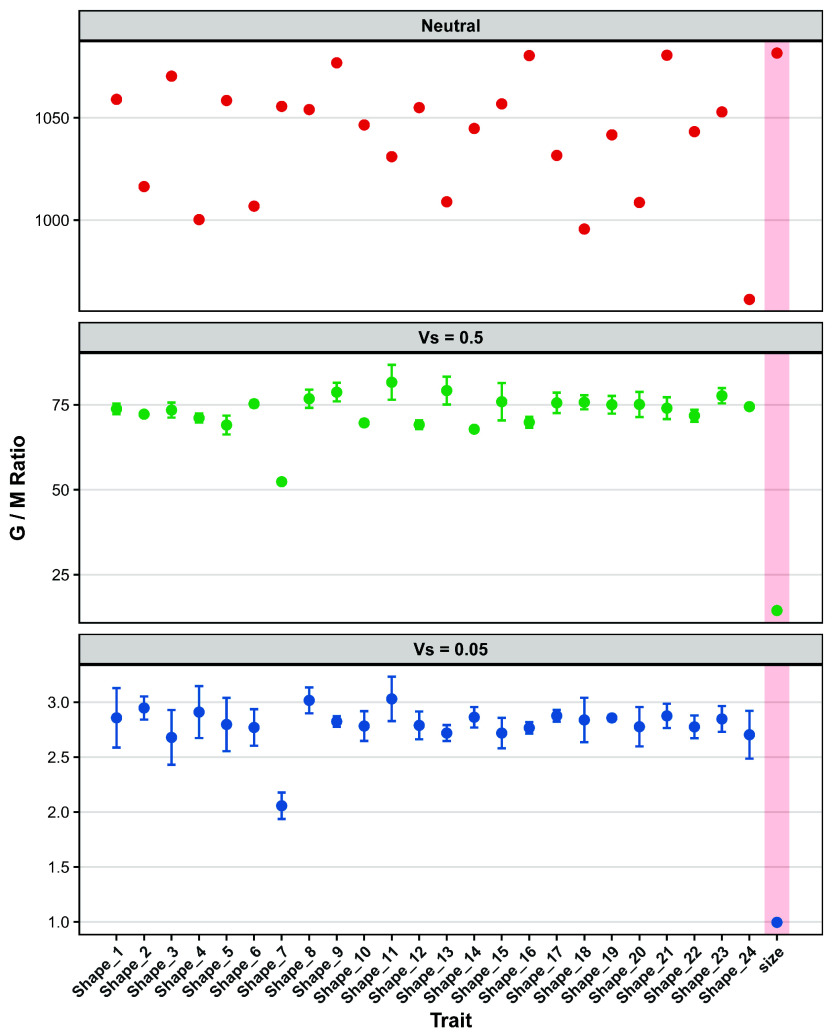
Ratio of genetic to mutational variance (G/M) across traits under varying evolutionary scenarios. Points represent the mean G/M ratio across replicates for each trait under pure neutral drift, weak stabilizing selection on the primary trait ($V_{s}=0.5$), and strong stabilizing selection on the primary trait ($V_{s}=0.05$). Error bars indicate ±1 SE of the mean across independent simulation replicates (n=3 for selection scenarios). The neutral panel reflects a single baseline simulation (n=1). The focal “size” trait is highlighted with a red shaded background. Note that the y-axis scales vary independently across panels to accommodate differing magnitudes of G/M ratios between scenarios.

Results from individual-based simulations support this expectation from the model (Fig. 3). Under completely neutral evolution, the G/M ratio is approximately uniform across all traits, including size, and hovers around ∼1,000—closely matching the theoretical expectation of $G/M=2N_{e}=2\times 500=1,000$ under pure drift. Under the single-axis selection model in which selection targets size, the G/M ratio for size is markedly reduced compared to shape traits, consistent with selection selectively eroding genetic variance along the selected axis. Furthermore, this G/M ratio profile is diagnostic of which trait is under direct selection: When the simulation is repeated with selection on a shape trait instead of size, the lowest G/M ratio shifts to that shape trait (*SI Appendix*, section C and Fig. S8).

### Reduced Rate of Shape Divergence Under Single-Axis Selection.

The alignment between *M*, *G*, and *R* alone does not distinguish single-axis selection from completely neutral evolution: Under complete neutrality, divergence would also scale with mutational variance (9, 14). The critical observation that requires explanation is that wing shape evolves orders of magnitude more slowly than expected from the abundant genetic variation available.

**Fig. 4.**
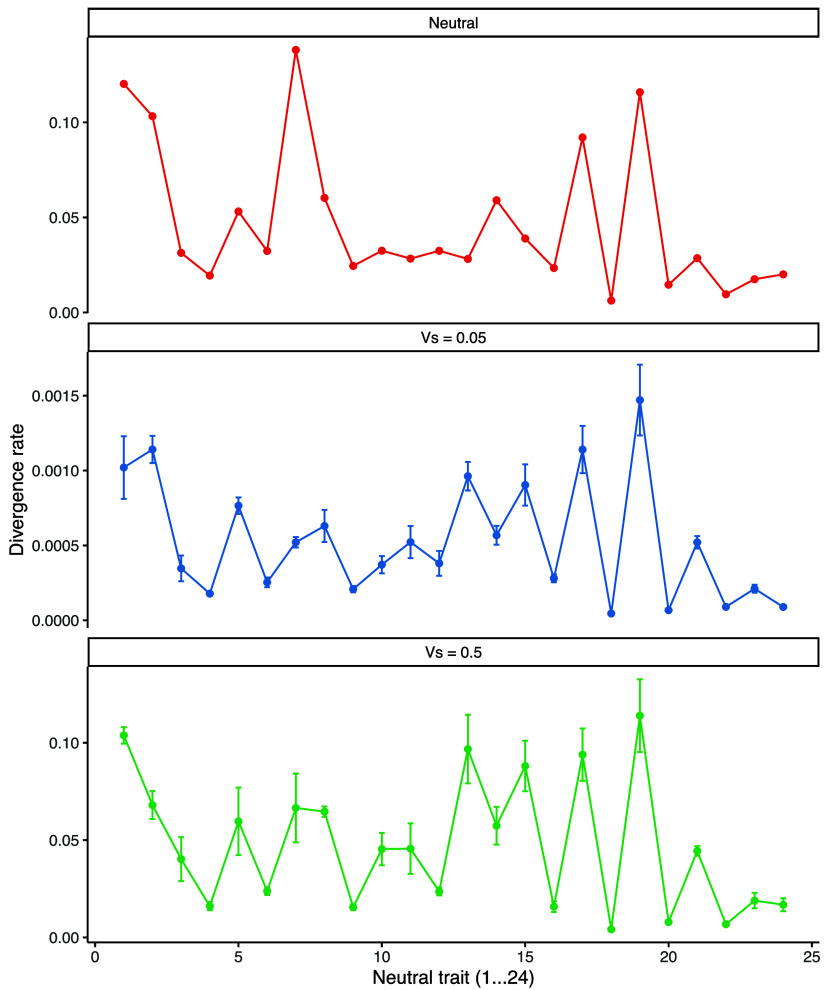
Divergence rate for 24 nonselected wing shape traits under single-axis selection. Each point represents the divergence rate (increase in among-population variance per generation) for one wing shape trait. Under strong selection on wing size ($V_{s}=0.05$, blue), divergence rates are substantially reduced compared to weak selection ($V_{s}=0.5$, green) and the completely neutral scenario (red, where both wing size and shape are neutral). Simulations used σ=0.0001, N=500, and 20 replicate populations evolved for 20,000 generations. Three independent simulation replicates were used for each $V_{s}$ condition ($V_{s}=0.05$ and $V_{s}=0.5$). Note that y-axis scales differ among panels.

**Fig. 5.**
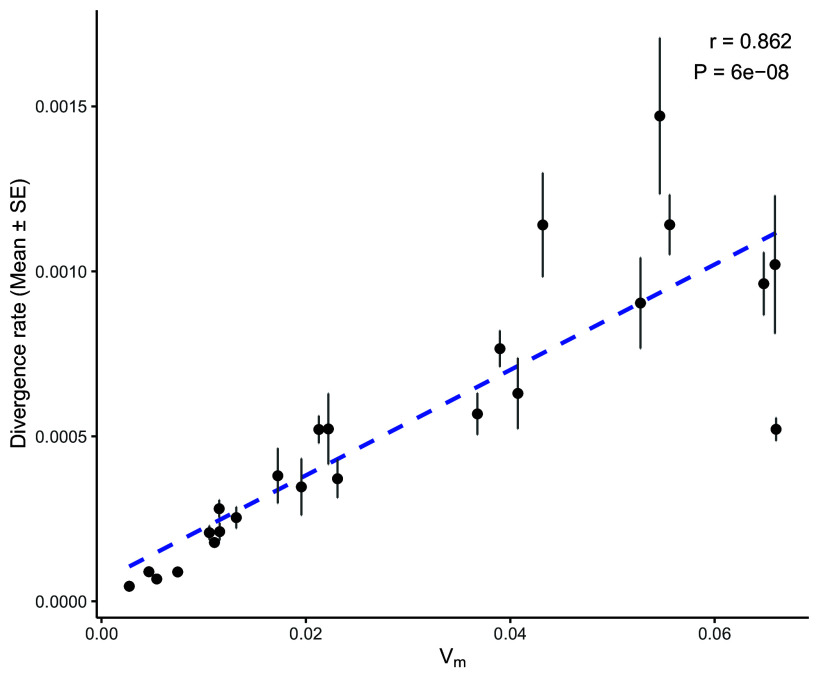
Divergence rate for nonselected traits scales with mutational variance under single-axis selection. Each point represents one of the 24 shape traits. The positive relationship between mutational variance ($V_{M}$) and divergence rate shows that traits with higher mutational input diverge faster, even when most traits are not under direct selection. Simulations used $V_{s}=0.05$ on wing size, σ=0.0001, N=500, and 20 replicate populations. Three independent simulation replicates were used.

Under the single-axis selection model, nonselected traits experience apparent selection through their mutational covariance with the primary target of selection (16, 17). Using a simplified two-trait model, I show that selection on the primary trait constrains the drift of correlated neutral traits by reducing their genetic variance, thereby reducing the total variance available for drift (see *SI Appendix*, section A for details). Simulation results using the full empirical *M* matrix support these predictions. Here, I use the term “divergence rate” to describe the rate of increase in among-lineage variance for these shape traits due to indirect selection responses, mutation, and drift. Fig. 4 shows the divergence rate for all nonselected wing shape traits under single-axis selection targeting size. Under stronger stabilizing selection on wing size ($V_{s}=0.05$), the divergence rate of every shape trait is substantially reduced (by approximately two orders of magnitude) compared to both weak selection ($V_{s}=0.5$) and the completely neutral scenario. Analogous patterns emerge in the two-trait toy model, where varying selection strength systematically modulates the effective divergence rate (*SI Appendix*, Fig. S3). Moreover, the relationship between mutational variance and divergence rate across traits (Fig. 5) shows that nonselected traits with higher mutational input diverge faster, preserving the rate–variance alignment.

### Single-Axis Selection and Correlational Selection Leave Distinct Imprints on *G*.

The single-axis selection model and the correlational selection hypothesis make distinct predictions about the eigenvalue structure of *G* relative to *M*. If correlational selection is the primary force shaping genetic covariances—favoring specific trait combinations that mirror the mutational architecture—then the equilibrium *G* should preserve the eigenvalue structure of *M*, maintaining similar eccentricity (i.e., similar ratios among eigenvalues). Conversely, under single-axis selection, where only a single primary trait is under selection, variance along the leading eigenvector of *M* (which is size-dominated) should be preferentially eroded, while variance in orthogonal directions remains largely unconstrained. This predicts that *G* should be less eccentric than *M* (here, size is included in the matrix comparisons).

Ref. 13 found that mutational correlations among fly wing traits are consistently stronger than the corresponding additive genetic correlations. Furthermore, mutational variance is highly concentrated along a single multivariate axis (high eccentricity), while genetic variance is more evenly distributed across axes (lower eccentricity). Reanalysis of *G* and *M* from ref. 9 reveals a similar pattern: The ratio of the first eigenvalue is substantially larger for *M* than for *G* (*SI Appendix*, Fig. S4 and section B3). These independent datasets converge on a consistent finding: Selection reduces, rather than reinforces, the eccentricity imposed by mutational input.

To formally test these competing predictions, I conducted simulations comparing equilibrium *G* under two scenarios (using *M* obtained from ref. 13): 1) the single-axis selection model, where only the primary trait is under stabilizing selection; and 2) correlational selection, where the selection matrix *S* is proportional to $M^{-1}$, imposing multivariate stabilizing selection that favors the trait combinations encoded by mutational pleiotropy. Two quantitative diagnostics were applied to the simulated equilibrium *G* matrices (5 replicates per scenario) to formally discriminate the two hypotheses.

**Fig. 6.**
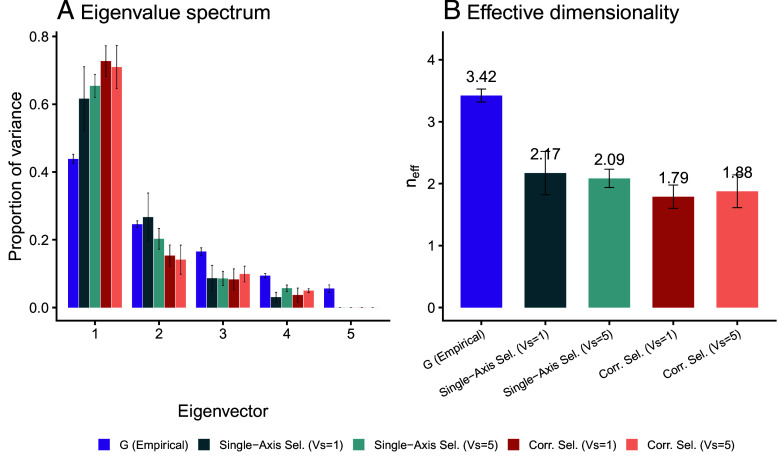
Eccentricity diagnostics for simulated and empirical *G* matrices. Two diagnostics applied to the empirical *G* from ref. 13 and equilibrium *G* under single-axis selection and correlational selection at two selection strengths ($V_{s}=1$ and $V_{s}=5$; 5 replicates each, N=500). (*A*) Eigenvalue spectrum showing the proportion of total genetic variance explained by each eigenvector. (*B*) Effective dimensionality ($n_{\text{eff}}$), measuring how evenly variance is distributed across eigenvectors. Error bars for simulated *G* show *±*1 s.d. across replicates; error bars for empirical *G* represent estimation uncertainty (parametric bootstrap from REML CIs reported in ref. 13).

The results (Fig. 6 and *SI Appendix*, Fig. S11) show that the eigenvalue spectrum of equilibrium *G* under single-axis selection is visibly less eccentric than that of *M*: the proportion of total genetic variance explained by the leading eigenvalue is reduced under single-axis selection, with the residual variance redistributed among the rest of the eigenvectors; in contrast, correlational selection preserves the eigenvalue proportions close to those of *M*. This flattening is also captured by the effective dimensionality of *G* ($n_{\text{eff}}$; refs. 18 and 19; see *Materials and Methods*): Under single-axis selection, $n_{\text{eff}}=2.17\pm 0.35$ at $V_{s}=1$ and 2.09±0.15 at $V_{s}=5$ (mean *±* s.d. across replicates), similar to empirical *G*, all exceeding the *M* baseline ($n_{\text{eff}}=1.80$, computed directly from the mutational covariance matrix), whereas correlational selection leaves $n_{\text{eff}}$ essentially unchanged (1.79±0.19 at $V_{s}=1$; 1.88±0.26 at $V_{s}=5$). These diagnostics collectively suggest that selection on a single trait reshapes the eigenvalue structure of *G* in a manner qualitatively distinct from multivariate correlational selection. Notably, the $n_{\text{eff}}$ of empirical *G* exceeds the simulated single-axis values at all selection strengths simulated (*SI Appendix*, Fig. S12), indicating that additional factors—such as environmental variance, selection on multiple targets, or more complex pleiotropic architectures—contribute to the dimensionality of empirical *G* beyond what the idealized single-trait model predicts.

These findings should be interpreted cautiously, given that empirically estimated *M* matrices may carry greater sampling uncertainty due to lower effective sample sizes (13), and the two scenarios tested here only represent idealized extremes of a continuum that may include both processes operating simultaneously.

## Discussion

The strong alignment between mutational variation (*M*), genetic variation (*G*), and species divergence (*R*) in fly wing shape has puzzled evolutionary biologists for nearly a decade. Here, I show that a minimal single-axis selection model—in which natural selection targets only one trait while all other wing traits evolve as correlated byproducts through within-module pleiotropy—is sufficient to reproduce these empirical patterns without invoking hidden constraints, correlational selection, or complex adaptive landscapes. The model predicts that the selected trait should exhibit the lowest G/M ratio, relative to other nonselected traits. Reanalysis of empirical *M* and *G* reveals that wing size may indeed be the primary target of selection among wing traits. Importantly, “single-axis selection” here refers to the effective dynamics within the wing trait module, not at the whole-organism level. Size need not be the only trait under selection across the entire phenotype, nor must it be the direct target of selection: Selection on wing size may itself be mediated by direct selection on correlated traits such as body mass, since the wing-size-to-body-mass ratio is a key determinant of flight performance (20). What matters for the present model is that, among all traits that experience selection, wing size exhibits the strongest and most stable pleiotropic connections with other wing shape dimensions, making it the trait whose selection—whether direct or indirect—is most crucial in producing the *M*–*G*–*R* alignment observed in fly wing shape. More broadly, these results illustrate that multivariate evolutionary patterns need not imply directly multivariate selection across every trait dimension. Ref. 3 showed that fluctuating multivariate optima can generate a positive relationship between evolvability and macroevolutionary divergence. The present model is complementary: It shows that comparable high-dimensional *M*–*G*–*R* alignment can also arise when the persistent component of selection is concentrated on one trait within a pleiotropically integrated module.

The results presented here focus on the *Drosophila* wing, but the underlying mechanism—apparent selection mediated by within-module pleiotropy—is not specific to this system. It should apply whenever mutations broadly affect multiple traits within an integrated developmental module. For example, macroevolutionary divergence in several systems predominantly tracks a single allometric line of least resistance, as documented in ruminant skulls (21), amphibian hindlimbs (22), and morphological differentiation in *Pristurus* geckos (23). If alignment can indeed arise from low-dimensional selection propagated through within-module pleiotropy, a clear prediction follows: the strength of alignment should depend on how pleiotropically integrated the measured traits are. Consistent with this logic, several key studies have shown that variance–divergence alignment is widespread beyond *Drosophila* (3, 24, 25), yet the strength of alignment varies considerably among systems with different trait compositions—a pattern that currently lacks a unifying explanation. The present model offers one plausible hypothesis: Trait sets composed entirely of morphological measurements within a single module should show stronger alignment than sets combining morphological and life-history traits. Testing this prediction across diverse taxa and trait modules would provide a powerful assessment of how broadly within-module pleiotropy contributes to observed micro–macro evolutionary alignments.

### Evidence for Wing Size as the Primary Selection Target.

The single-axis selection model and the empirical G/M analyses both point to wing size as the most likely dominant target of selection within the wing module. This expectation is biologically plausible because wing size directly affects aerodynamic performance, including lift generation and flight power requirements. Across flying animals, size-related wing traits are associated with multiple performance and fitness components, including mating success in insects (26–28), migration-related performance in monarch butterflies (29, 30), thermal properties (31), flight–reproduction trade-offs (32, 33), and broad-scale flight-efficiency gradients (34). More directly, a multivariate sexual-selection analysis of *D. melanogaster* found that directional selection strength on centroid size, derived from standard linear selection gradients, was stronger than selection on wing color or shape traits (28). These observations support the idea that some apparent selection on wing shape may arise indirectly through selection on size. However, this interpretation is not exclusive: Allometry-corrected shape selection can remain significant (27), and correlated size–shape variation makes it difficult to fully separate direct selection on size from direct selection on shape.

### Can Single-Axis Selection Fully Resolve the Rate Paradox?

Whether the proposed single-axis selection model can fully account for the slower-than-neutral divergence rate observed in wing shape depends on two key parameters: The strength of selection on wing size and the rate at which the size optimum diverges among lineages. Both parameters jointly determine how tightly shape evolution is leashed. Empirical estimates suggest that shape divergence proceeds at approximately $10^{-4}$ of the neutral expectation (9). In the present simulations (N=500, $V_{s}=0.05$), the correlated-response effect reduced the effective divergence rate by approximately two orders of magnitude relative to the neutral scenario (Fig. 4); however, this reduction reflects a single, arbitrary parameterization and should not be interpreted as the model’s upper bound—stronger selection or slower optimum divergence would yield correspondingly greater constraint. Therefore, whether selection on wing size is sufficiently strong in nature is also an empirical question that warrants direct investigation.

The single-axis selection model can be viewed as a refinement of the deleterious pleiotropy hypothesis of ref. 9. That hypothesis invokes pleiotropic effects on unmeasured traits outside the wing shape complex as the source of constraint. The present model suggests a more specific mechanism: The relevant pleiotropy may reside primarily within the wing module itself, where selection on size constrains shape evolution through within-module pleiotropic mutation. Pleiotropic effects on traits outside the wing module may provide additional constraint; indeed, the combined action of within-module apparent selection, large effective population size, and extramodular pleiotropic costs may together be needed to account for the extreme evolutionary stasis of wing shape.

### Stability of the Mutational Variance–Covariance Matrix.

A key assumption of the single-axis selection model is that the structure of *M* remains stable over macroevolutionary time. Several lines of evidence support this assumption. First, genetic variance–covariance matrices (*G*) appear similar between Sepsidae and *D. melanogaster*, which diverged approximately 64 Mya (10, 35). Common subspace analyses comparing *G* matrices across these taxa reveal substantial alignment regardless of whether *S. fulgens* or *S. punctum* is compared to *D. melanogaster* (*SI Appendix*, section D, Figs. S9 and S10). However, because *G* is determined by both mutational input and selection, observing that *G* is conserved does not strictly imply that *M* is conserved. If selection is strong and conserved, it could constrain *G* to remain stable even if the underlying mutational architecture fluctuates. Inferring that *M* is conserved from a conserved *G* requires the assumption that selection is not completely overriding the mutational variance. Second, the highly polygenic genetic architecture of wing shape traits (*SI Appendix*, section E and Table S2) may enhance the stability of *M* and *G* by buffering against the effects of individual mutations (36, 37). Nevertheless, direct measurement of *M* across divergent lineages remains an important goal for future research.

### Ultimate versus Proximate Causes.

It is important to distinguish between proximate and ultimate explanations for the *M*-*G*-*R* alignment. The single-axis selection model provides a proximate mechanism: Given a stable *M* matrix and selection on a single trait, alignment emerges as a natural consequence. Similarly, the multidimensional fluctuating-selection model (3) operates as an alternative proximate mechanism: Assuming a preexisting mutational and genetic architecture, it demonstrates how rapid fluctuations in optima produce macroevolutionary divergence (*R*) that aligns with *G*. However, the ultimate origin of the mutational architecture itself remains an open question. Mutation is evolvable (4, 38), and the structure of *M* may itself be shaped by selection over deep evolutionary time.

Several ultimate-level hypotheses offer explanations for the origin of *M*. The “moving corridor” hypothesis proposes that the mutational architecture itself evolves to align with the predominant direction of optimum movement: Lineages whose *M* channels variation along the historical axis of environmental change adapt more readily, so over deep time *M* comes to mirror the typical trajectory of shifting optima. Correlational selection models propose that persistent selection on trait combinations (e.g., allometric relationships) shapes *M* and *G* simultaneously.

## Materials and Methods

### Model Overview.

The single-axis selection model, proposed here, posits that natural selection targets only one primary trait, while remaining traits evolve as neutral byproducts through their mutational covariance with the selected trait. The instantaneous selection gradient *β* implied by the Gaussian fitness function is therefore:

$$\begin{array}{c} \beta ={\left[\beta_{s},0,0,\cdots ,0\right]}^{T}, \end{array}$$

where $\beta_{s}$ reflects the strength of selection on the primary trait, determined by the distance between the population mean and the current optimum. The population divergence in the primary trait arises from lineage-specific moving optima. In this reduced wing-only model, the dependence of optimal wing size on other traits (e.g., body mass) and environmental conditions is represented as a lineage-specific moving optimum for wing size. Under this formulation, wing shape traits are effectively neutral, but experience indirect evolutionary change through their mutational covariance with the selected trait.

### Individual-Based Simulations.

I assessed the single-axis selection model using individual-based simulations. I simulated populations of N=500 hermaphroditic, sexually reproducing individuals with nonoverlapping generations. Phenotypes were determined by n=50 unlinked diploid loci, with alleles assumed to be fully pleiotropic (i.e., affecting all phenotypic traits). The life cycle proceeded in three discrete steps:

1. **Phenotype and fitness assignment.** Each individual’s phenotypic value for every trait was obtained by summing allelic breeding values across all loci, assuming strict additivity (no dominance or epistasis at the phenotypic level, though epistasis may arise at the fitness level). Fitness was then assigned according to a Gaussian stabilizing selection function applied solely to the primary trait:
  $$\begin{array}{c} W\left(z_{1}\right)=\;\exp\left(-\frac{{\left(z_{1}-\theta \right)}^{2}}{2V_{s}}\right), \end{array}$$
  where $z_{1}$ is the individual’s primary trait value, *θ* is the current phenotypic optimum, and $V_{s}$ controls the width of the fitness peak (smaller $V_{s}$ imposes stronger selection).
2. **Selection and mating.** Parents were sampled with replacement in proportion to their fitness, and two distinct individuals were paired to produce each offspring (selfing was excluded). This fitness-weighted sampling was repeated *N* times per generation to maintain constant population size.
3. **Recombination and mutation.** Offspring genotypes were assembled by independently drawing one allele per locus from each parent, corresponding to free recombination among all loci. Mutations occurred at a rate of μ=0.01 per allele per generation (genomic mutation rate U=1 per individual per generation). Pleiotropic mutational effects were drawn from a multivariate normal distribution with variance–covariance matrix M, estimated empirically under homozygous conditions by ref. 9.

Each simulation began with a burn-in period of 10N generations, with the optimum set to zero throughout. Following burn-in, 20 replicate populations were evolved for T=20,000 generations under stabilizing selection on lineage-specific moving optima for the primary trait, where each replicate was assigned an optimum moving rate sampled from a normal distribution with mean μ=0 and SD σ=0.0001. Selection strength was varied across simulations by setting $V_{s}=0.05$ (strong selection) or $V_{s}=0.5$ (weak selection).

The within-population genetic variance–covariance matrix G was computed as the phenotypic covariance matrix within each replicate population at the final generation and then averaged across all 20 replicates. The among-population divergence matrix R was computed as the covariance matrix of the 20 replicate-population trait means.

### Comparing Variance–Covariance Matrices.

To quantify the alignment between mutational variance (M), genetic variance (G), and evolutionary divergence (R), I employed the common subspace analysis of ref. 14. This approach compares matrices along a shared set of orthogonal axes defined by the eigenvectors of M.

Specifically, I decomposed the homozygous mutational variance–covariance matrix ($M_{\mathrm{hom}}$) estimated in *D. melanogaster* into its eigenvector matrix K. For each variance–covariance matrix of interest ($X\in \left\{G,R\right\}$), I calculated the variance along each eigenvector as the diagonal entries of $K^{T}XK$. I then regressed $\log_{10}$-transformed variances in R or G against $\log_{10}$-transformed variances in M using ordinary least squares (OLS), obtaining regression slopes and coefficients of determination ($R^{2}$). To avoid comparisons along dimensions with negligible variance, analyses were restricted to the first 18 eigenvectors of M.

### Eccentricity Diagnostics.

To discriminate between the single-axis selection and correlational selection hypotheses, I applied two quantitative diagnostics to the eigenvalue structure of the equilibrium genetic variance–covariance matrix *G* relative to the mutational variance–covariance matrix *M*.

The first diagnostic is the eigenvalue spectrum, which compares the proportion of total genetic variance captured by each successive eigenvector of *G*. Under single-axis selection, selection on the primary trait preferentially erodes variance along the leading eigenvector of *M* (which is dominated by size), redistributing variance toward trailing eigenvectors and flattening the spectrum. Under correlational selection, the eigenvalue proportions of *G* should remain similar to those of *M*.

The second diagnostic is the effective dimensionality, which is defined as participation ratio (18, 19):

$$n_{\text{eff}}=\frac{{\left(\sum_{i}\lambda_{i}\right)}^{2}}{\sum_{i}\lambda_{i}^{2}},$$

where $\lambda_{i}$ are the eigenvalues of *G*. This index measures how evenly variance is distributed across eigenvectors: $n_{\text{eff}}=1$ when all variance is concentrated along a single axis, and $n_{\text{eff}}=n$ when variance is uniformly distributed across all *n* eigenvectors. This definition of effective dimensionality is different from the one used in ref. 39. Yet, all the results for $n_{\text{eff}}$ are qualitatively similar regardless of which definition is used. Single-axis selection is predicted to increase $n_{\text{eff}}$ above the *M* baseline by reducing the dominance of the leading eigenvalue.

For each scenario, equilibrium *G* matrices were obtained from individual-based simulations using the empirical *M* matrix of ref. 13 (5 traits: centroid size plus 4 shape variables). The empirical *M* was forced to be positive semidefinite prior to use. Simulations were run with N=500 individuals for 5 independent replicates per scenario. Because the ref. 13 traits are on a different measurement scale (due to different traits and scaling methods during analyses) from those of ref. 9, the stabilizing selection width $V_{s}$ was adjusted. For the empirical *G* matrix (ref. 13), estimation uncertainty was quantified by parametric bootstrap: 200 replicate matrices were generated by sampling each element from a normal distribution centered on the point estimate with SD equal to the reported SE, followed by symmetrization and projection onto the nearest positive semidefinite matrix.

## Supplementary Material

Appendix 01 (PDF)

## Data Availability

All simulation code, analysis scripts, and data files necessary to reproduce the main figures are publicly available at https://github.com/haorancai/flywingmodel. Previously published data were used for this work (9, 13).
