# Supplementary material for: Persistent selection on size explains micro- and macroevolutionary alignments in fly wings
Source: Proc Natl Acad Sci U S A. 2026 Jun 18;123(25):e2612940123. doi: 10.1073/pnas.2612940123 (PMC13291493; doi:10.1073/pnas.2612940123)
Supplement: Supplementary file 1 — Appendix 01 (PDF) [file pnas.2612940123.sapp.pdf]

Supporting Information for

Persistent selection on size explains micro- and macroevolutionary alignments in fly wings

Haoran Cai

To whom correspondence should be addressed. E-mail: hrcai@ucla.edu

This PDF file includes:

Supporting text
Figs. S1 to S12
Tables S1 to S2
SI References

Supporting Information Text

Supplementary Materials: Table of Contents

Appendix A. Trait Mean Dynamics under Apparent Stabilizing Selection ... 2
A.1 Deterministic dynamics ... 3
A.2 Reduced genetic drift due to genetic correlation ... 3
A.3 Numerical simulation ... 4
A.4 Generality: moving and fluctuating optima ... 7
A.5 Effect of unequal mutational variance: does alignment between M and selection matter? ... 8

Appendix B. Expected G under mutation–selection balance ... 8
B.1 Traditional Framework: Equilibrium in Infinite Populations ... 8
B.2 Traditional Framework: Finite Population with Drift ... 9
B.3 Interpreting empirical measurements of G and M ... 9

Appendix C. Robustness of alignment among G, R, and M ... 11
C.1 Sensitivity to selection strength and reference matrix ... 11
C.2 Selection on a low-variance shape trait: full 25-trait robustness test ... 12

Appendix D. Comparison of G Between *Sepsis* and *Drosophila* ... 15

Appendix E. Polygenic basis of fly wing shape and vein positioning ... 17

Appendix F. Eccentricity analysis — supplementary figures ... 18

Appendix A: Trait Mean Dynamics under Apparent Stabilizing Selection

The single-axis selection model assumes that selection acts directly only on a primary trait, while other traits are neutral. However, these neutral traits do not exhibit pure Brownian motion. Instead, they experience apparent stabilizing selection due to pleiotropic effects associated with the selected trait (1–6)—individuals carrying more deleterious alleles also tend to have more extreme (positive and negative) trait values, generating a quadratic relationship between trait value and fitness, and thus a spurious signature of stabilizing selection.

I consider two quantitative traits, A and B, with mean breeding values

$$\bar{\mathbf{z}}_t = \begin{pmatrix} \bar{z}_{A,t} \\ \bar{z}_{B,t} \end{pmatrix}.$$

Trait A is subject to Gaussian stabilizing selection, while trait B has no direct effect on fitness:

$$W(z_A, z_B) = \exp\left[-\frac{(z_A - \theta)^2}{2V_s}\right], \tag{1}$$

with optimum θ and V_s measures the strength of stabilizing selection. Trait B does not appear in W, so it has no direct effect on fitness.

The directional selection gradient (Lande–Arnold) at the mean is

$$\boldsymbol{\beta} = \frac{\partial \ln W}{\partial \boldsymbol{z}}\bigg|_{\bar{\boldsymbol{z}}} = \begin{pmatrix} -\dfrac{\bar{z}_A - \theta}{V_s} \\ 0 \end{pmatrix} = \begin{pmatrix} \beta_A \\ \beta_B \end{pmatrix}. \tag{2}$$

Let the additive genetic covariance matrix be

$$G = \begin{pmatrix} G_{AA} & G_{AB} \\ G_{AB} & G_{BB} \end{pmatrix}. \tag{3}$$

In an infinite population (no drift), the multivariate breeder's equation gives

$$\Delta \bar{\boldsymbol{z}}_{\text{sel}} = G\boldsymbol{\beta}. \tag{4}$$

For a finite population of effective size N_e, we add a drift term $\boldsymbol{\varepsilon}$:

$$\Delta \bar{\boldsymbol{z}} = G\boldsymbol{\beta} + \boldsymbol{\varepsilon}, \tag{5}$$

with

$$\mathbb{E}[\boldsymbol{\varepsilon}] = \mathbf{0}, \qquad \text{Cov}(\boldsymbol{\varepsilon}) = \frac{G}{2N_e}. \tag{6}$$

Component-wise:

$$\Delta \bar{z}_A = -\frac{G_{AA}}{V_s}(\bar{z}_A - \theta) + \varepsilon_A, \tag{7}$$

$$\Delta \bar{z}_B = -\frac{G_{AB}}{V_s}(\bar{z}_A - \theta) + \varepsilon_B, \tag{8}$$

$$\mathrm{Var}(\varepsilon_A) = \frac{G_{AA}}{2N_e}, \quad \mathrm{Var}(\varepsilon_B) = \frac{G_{BB}}{2N_e}, \quad \mathrm{Cov}(\varepsilon_A, \varepsilon_B) = \frac{G_{AB}}{2N_e}. \tag{9}$$

A.1 Deterministic dynamics. Let $m_A(t) = \mathbb{E}[\bar{z}_A(t)]$, $m_B(t) = \mathbb{E}[\bar{z}_B(t)]$. Then

$$\frac{dm_A}{dt} = -\frac{G_{AA}}{V_s}\big(m_A - \theta\big), \tag{10}$$

$$\frac{dm_B}{dt} = -\frac{G_{AB}}{V_s}\big(m_A - \theta\big). \tag{11}$$

The solution for m_A is

$$m_A(t) = \theta + \big(m_A(0) - \theta\big)e^{-(G_{AA}/V_s)t}. \tag{12}$$

Using this in dm_B/dt:

$$\frac{dm_B}{dt} = \frac{G_{AB}}{G_{AA}}\frac{dm_A}{dt}, \tag{13}$$

so integrating,

$$m_B(t) = m_B(0) + \frac{G_{AB}}{G_{AA}}\big(m_A(t) - m_A(0)\big). \tag{14}$$

As $t \to \infty$, $m_A(t) \to \theta$ and

$$\boxed{\begin{aligned} &\mathbb{E}[\bar{z}_A(\infty)] = \theta, \\ &\mathbb{E}[\bar{z}_B(\infty)] = m_B(0) + \frac{G_{AB}}{G_{AA}}\big(\theta - m_A(0)\big). \end{aligned}} \tag{15}$$

Therefore, when assuming constant $\mathbf{G}$ the expected mean of trait A (selected trait) goes to the optimum while the expected mean of trait B (neutral trait) goes to some constant (deterministic transient displacement) determined both by the initial mean of trait B and how far A had to move to reach the optimum, scaled by G_{AB}/G_{AA}.

A.2 Reduced genetic drift due to genetic correlation. While the deterministic means follow the dynamics above, in any single finite population the realized means fluctuate around those expectations. I now show that, near the deterministic equilibrium, mutational correlation with a selected trait constrains the rate of neutral drift.

Assumption. Throughout this subsection, I treat G as constant. In reality, G is itself shaped by the balance of mutation, selection, and drift (see Appendix B). Because G evolves on a timescale comparable to that of the mean dynamics, the formula derived below should be viewed as an instantaneous diffusion rate conditioned on the current value of G, rather than a long-run prediction. Section A.3 compares this instantaneous prediction with full simulations that allow G to evolve.

Coordinate rotation. Define the regression coefficient of trait B on trait A:

$$k = \frac{G_{AB}}{G_{AA}}, \tag{16}$$

and introduce the rotated coordinates

$$x = \bar{z}_A, \qquad y = \bar{z}_B - k\,\bar{z}_A. \tag{17}$$

The coordinate x captures the selected trait directly, while y isolates the component of trait B that is genetically independent of trait A—that is, the residual after regressing out the correlated response. For any generic selection gradient β_A acting exclusively on trait A (i.e., $\boldsymbol{\beta} = [\beta_A, 0]^\mathsf{T}$), the component-wise changes in the means are $\Delta\bar{z}_A = G_{AA}\beta_A + \varepsilon_A$ and $\Delta\bar{z}_B = G_{AB}\beta_A + \varepsilon_B$. The change in y per generation is therefore

$$\begin{aligned} \Delta y &= \Delta\bar{z}_B - k\,\Delta\bar{z}_A \\ &= \Big[G_{AB}\beta_A + \varepsilon_B\Big] - k\Big[G_{AA}\beta_A + \varepsilon_A\Big] \\ &= \beta_A\big(G_{AB} - k\,G_{AA}\big) + (\varepsilon_B - k\,\varepsilon_A) \\ &= \varepsilon_B - k\,\varepsilon_A, \end{aligned} \tag{18}$$

where the selection terms cancel exactly for any β_A because $k = G_{AB}/G_{AA}$. Thus y is a pure random walk regardless of the functional form of selection on trait A.

The drift noise in the original coordinates has covariance

$$\mathrm{Cov}(\boldsymbol{\varepsilon}) = \frac{G}{2N_e}.$$

Defining $\varepsilon_y = \varepsilon_B - k\,\varepsilon_A$, we compute

$$\begin{aligned}\mathrm{Var}(\varepsilon_y) &= \frac{1}{2N_e}\big(G_{BB} + k^2 G_{AA} - 2k\,G_{AB}\big)\\ &= \frac{1}{2N_e}\left(G_{BB} - \frac{G_{AB}^2}{G_{AA}}\right)\\ &= \frac{G_{BB}}{2N_e}(1-\rho_G^2), \end{aligned} \qquad [19]$$

where $\rho_G = G_{AB}/\sqrt{G_{AA}G_{BB}}$ is the genetic correlation. The drift increments ε_x and ε_y are uncorrelated ($\mathrm{Cov}(\varepsilon_x, \varepsilon_y) = 0$), confirming that y evolves independently of the selected coordinate x.

Since Δy is a mean-zero random variable with per-generation variance $\mathrm{Var}(\varepsilon_y)$, the among-lineage variance grows linearly in time:

$$\mathrm{Var}[y(t)] = D_y\,t, \qquad D_y \equiv \frac{G_{BB}}{2N_e}(1-\rho_G^2).$$

I refer to D_y as the effective drift rate (per-generation rate of increase in among-lineage variance):

$$D_y = \frac{G_{BB}}{2N_e}(1-\rho_G^2). \qquad [20]$$

Eq. 20 shows that the effective drift rate of trait B depends on the conditional genetic variance $G_{BB|A} = G_{BB}(1-\rho_G^2)$—the fraction of genetic variance in B that is independent of the selected trait.

A.3 Numerical simulation. To assess how selection strength on trait A constrains the drift of trait B, I conducted simulations under the Gaussian regime using the two-trait model described above. Note that the simulations in this section span a range of selection strengths ($V_s = 0.001$ to 0.5); the lower end of this range represents a strong selection regime where selection is the dominant force shaping the equilibrium G matrix.

Variance erosion (G_{BB}). As shown in Fig. S2, stronger selection on trait A (smaller V_s) reduces the equilibrium genetic variance of the neutral trait G_{BB}, consistent with apparent stabilizing selection acting on trait B. This occurs because mutations that affect both traits are eliminated by selection on A, effectively eroding genetic variance in B even though B is not directly selected. This variance erosion is the primary mechanism by which single-axis selection constrains neutral drift.

Behavior of ρ_G. In the strong selection regime shown in Fig. S1, the equilibrium genetic correlation ρ_G between traits A and B increases toward ρ_M as selection strengthens (smaller V_s). However, across a broader range of selection strengths, the relationship between V_s and ρ_G is non-monotonic: under weak selection $\rho_G \approx \rho_M$ (mutation-dominated equilibrium), at moderate selection strengths ρ_G can decrease below ρ_M as selection differentially erodes G matrix components, and under very strong selection ρ_G rebounds toward ρ_M.

Effective population size reduction. Strong selection creates unequal reproductive success, reducing the effective population size N_e below the census size (7, 8). Since the drift rate scales as $1/(2N_e)$, this reduction partially *counteracts* the drift-constraining effect of variance erosion. For $V_s = 0.05$, N_e decreases modestly (from approximately 423 to 390, ~8%); only under extreme selection ($V_s = 0.001$) does N_e drop dramatically (to ~9), making this secondary effect quantitatively important.

Combined effect on drift. The realized trajectories of trait B across 20 replicate populations (Fig. S3) directly show that the net outcome is drift constraint: effective drift rates decrease with stronger selection and higher mutational correlation, confirming that variance erosion through apparent stabilizing selection is the dominant force.

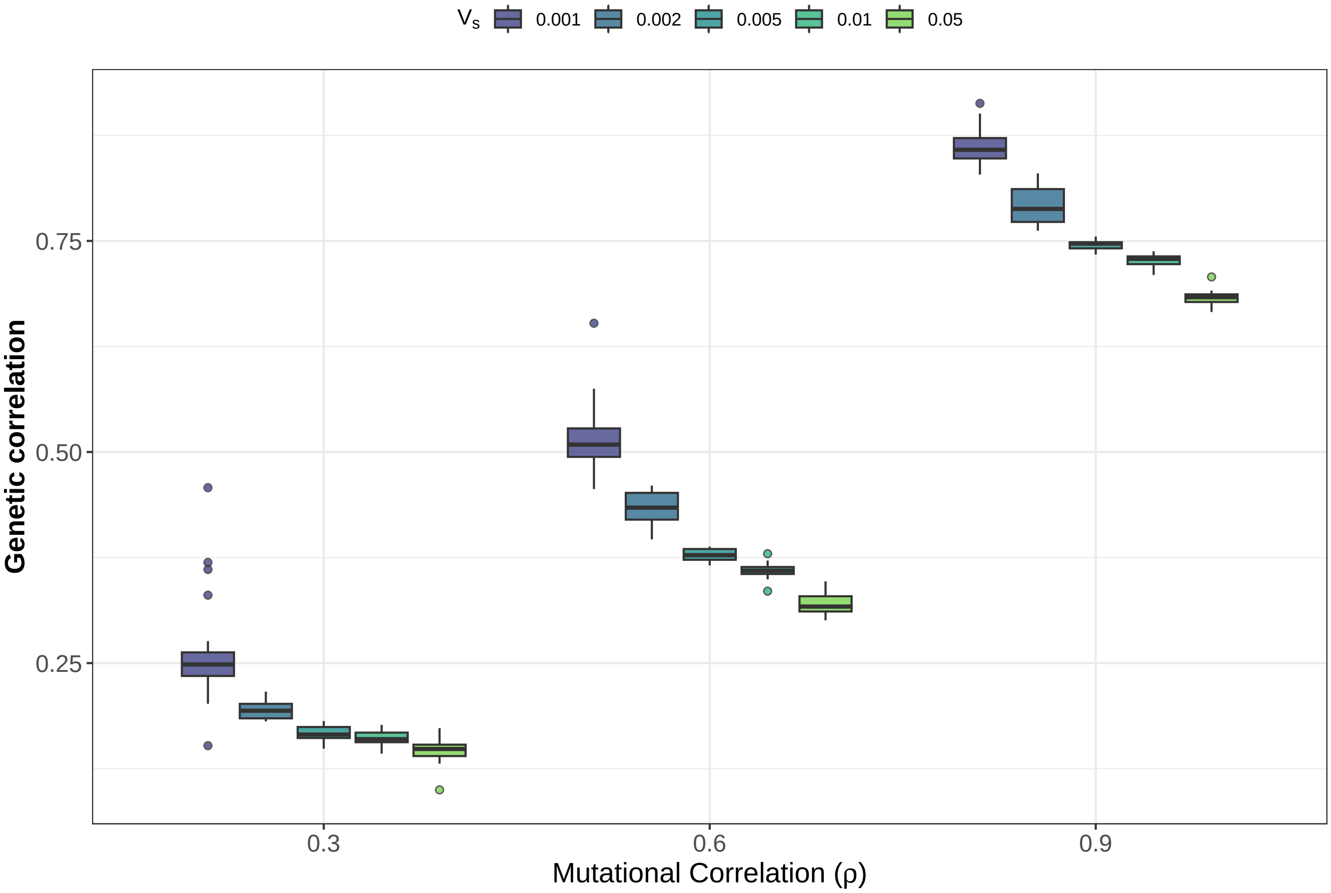

Fig. S1. Genetic correlation responds to selection strength under single-axis selection. Equilibrium genetic correlation (ρ_G) between the selected trait (A) and neutral trait (B) as a function of mutational correlation and selection strength (V_s). Mean ρ_G over the final 2,000 of 10,000 generations is shown. In the strong selection regime shown here, ρ_G approaches ρ_M as V_s decreases. However, across a broader range of selection strengths the relationship is non-monotonic: ρ_G is lowest at moderate V_s and rebounds toward ρ_M under both weak selection (where G is mutation-dominated) and very strong selection (where G_{AA} is severely depleted). Simulations: $N = 500$, 100 loci, $\mu = 0.01$, $V_\alpha = 0.0025$, 20 replicates per condition.

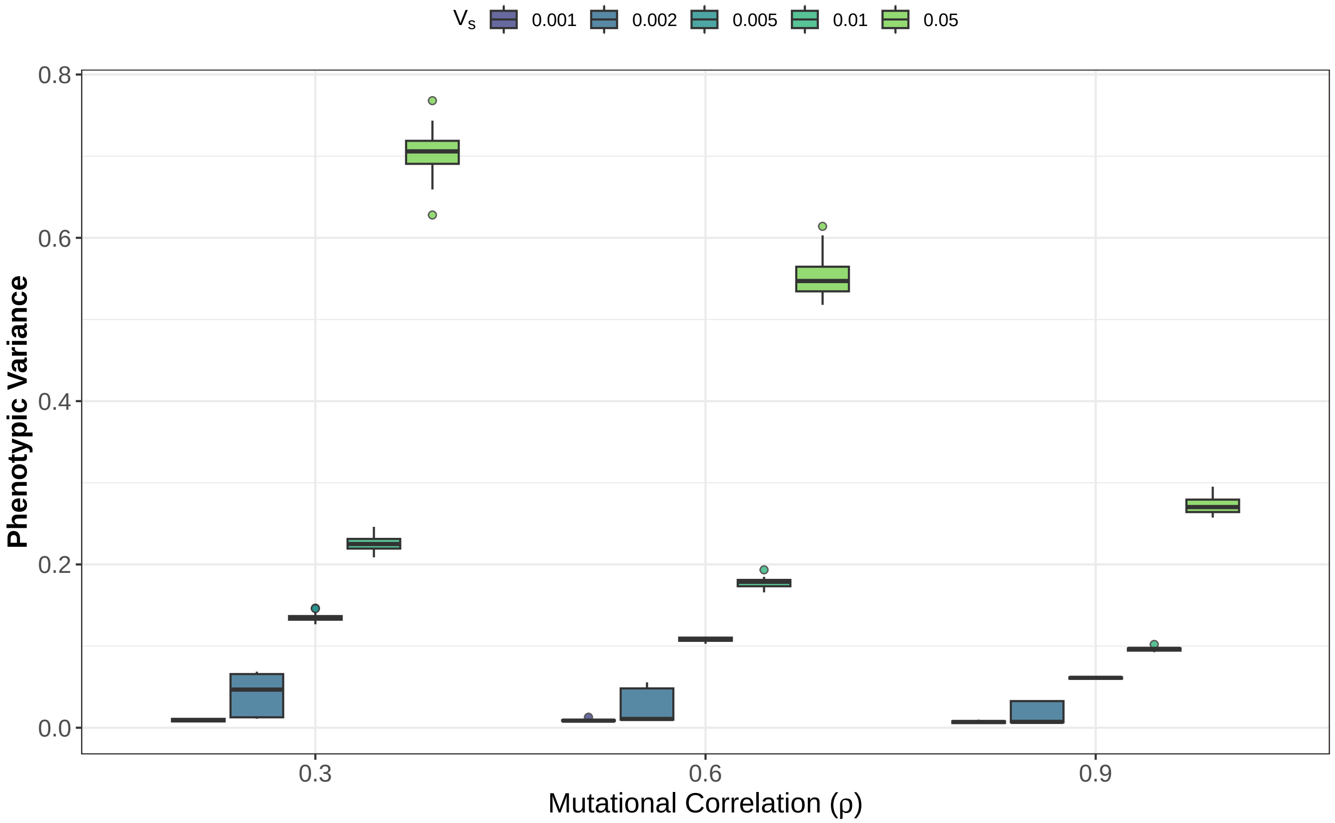

Fig. S2. Genetic variance of the neutral trait decreases with selection strength. Equilibrium genetic variance (G_{BB}) of the neutral trait (B) as a function of mutational correlation and selection strength on trait A. Stronger selection on the primary trait reduces genetic variance in correlated neutral traits through apparent stabilizing selection. Mean G_{BB} over the final 2,000 of 1,0000 generations. Simulations: $N = 500$, 100 loci, $\mu = 0.01$, $V_\alpha = 0.0025$, 20 replicates per condition.

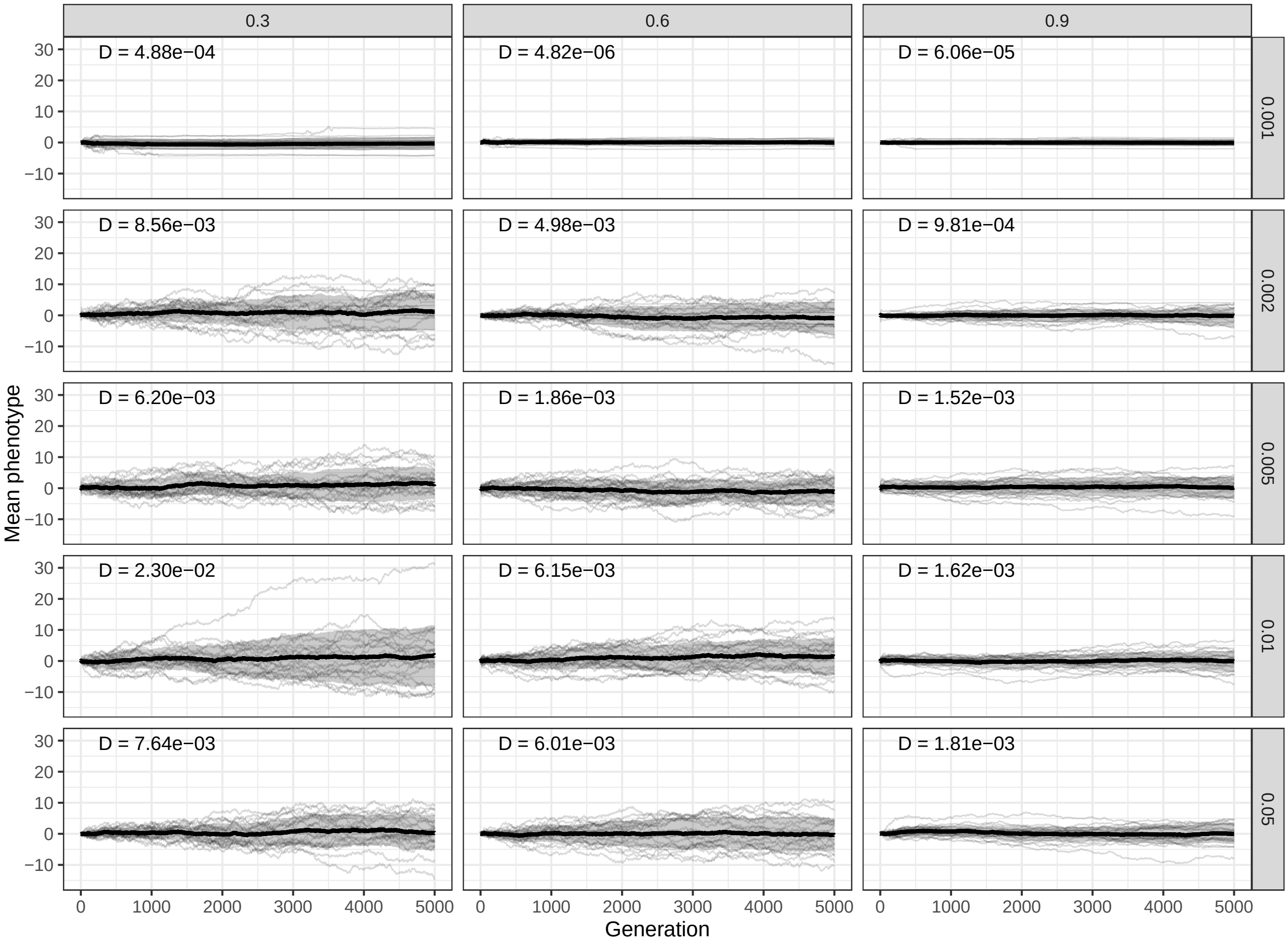

Fig. S3. Drift trajectories of the neutral trait under varying selection strengths and mutational correlations. Each panel shows mean trajectories (bold lines) and each realized trajectory across 20 replicate populations over 5,000 generations. Columns represent three levels of mutational correlation (ρ_M) between selected trait A and neutral trait B; rows show varying selection strengths on A. D indicates the effective drift rate (variance increase per generation). Stronger selection and higher mutational correlation increasingly constrain drift of the neutral trait. Simulations: $N = 500$, 100 loci, $\mu = 0.01$, $V_\alpha = 0.0025$.

A.4 Generality: moving and fluctuating optima. The drift-reduction result derived in Section A.2 assumed a fixed optimum θ, but the main-text simulations use a moving optimum. The result generalizes directly. When $\theta = \theta(t)$, the component-wise dynamics become

$$\Delta \bar{z}_A = -\frac{G_{AA}}{V_s}\big(\bar{z}_A - \theta(t)\big) + \varepsilon_A, \qquad [21]$$

$$\Delta \bar{z}_B = -\frac{G_{AB}}{V_s}\big(\bar{z}_A - \theta(t)\big) + \varepsilon_B. \qquad [22]$$

In the rotated coordinate $y = \bar{z}_B - k\,\bar{z}_A$ (Section A.2), the selection terms cancel exactly as before:

$$\Delta y = \varepsilon_B - k\,\varepsilon_A, \qquad \mathrm{Var}(\Delta y) = \frac{G_{BB}}{2N_e}(1 - \rho_G^2) = D_y, \qquad [23]$$

identical to the fixed-optimum case (Eq. 20). The form of $\theta(t)$—whether fixed, linearly moving, or stochastically fluctuating—determines the deterministic trajectory of trait B through its correlated response $k\,\bar{z}_A(t)$, but the stochastic drift component y is invariant.

A subtler concern is whether a moving optimum alters the equilibrium G matrix itself, which would indirectly change D_y through changes in G_{BB} and ρ_G. Under classical lag-load theory (9, 10), a slowly moving optimum (with rate $\kappa \ll \kappa_c$, where κ_c is the critical rate of environmental change) produces a small, constant lag, and the equilibrium G remains close to its stationary mutation–selection–drift value. In the main-text simulations, the optimum velocities v_i are drawn from a distribution with small σ, placing the system well within the slow-movement regime. More importantly, all simulation results are computed from G matrices measured directly under the full moving-optimum protocol, not from fixed-optimum analytical approximations.

A.5 Effect of unequal mutational variance: does alignment between M and selection matter? A potential concern with the single-axis selection model is that the selected trait (wing size) happens to carry the greatest mutational variance. To test whether the model's ability to constrain neutral traits depends on this alignment, I examine what happens when stabilizing selection instead targets the trait with the least mutational variance.

Using the two-trait simulation framework described above, I set the per-locus mutational effect variance of trait A to be 100× larger than that of trait B ($V_{\alpha,A} = 0.0025$, $V_{\alpha,B} = 2.5 \times 10^{-5}$), with mutational correlation $\rho_M = 0.9$.

Two scenarios are compared:

1. **Selection on the high-V_M trait** (trait A): stabilizing selection targets the trait with greater mutational variance, mimicking the empirical situation in which wing size has the largest V_M.

2. **Selection on the low-V_M trait** (trait B): stabilizing selection targets the trait with 100× smaller mutational variance.

For each scenario, I simulated two selection strengths ($V_s = 0.005$ and $V_s = 0.1$) alongside a fully neutral baseline (no selection on either trait). Each condition was replicated across 20 independent populations of $N = 500$ diploid individuals evolving for 2000 generations (100 loci, $\mu = 0.01$ per locus per generation).

Because the two traits have very different mutational inputs, I quantify the effective constraint on the nonselected trait by the *drift rate ratio*: the rate at which among-replicate variance of the nonselected trait mean increases, normalized by the corresponding rate under the neutral baseline. A ratio of 1 means no constraint (drift as fast as neutral); a ratio near 0 means strong constraint.

Results. Table S1 summarizes the results across 20 replicates per condition. The results show that both scenarios constrain the nonselected trait. In every selection condition, the drift rate ratio is less than 1 (Table S1), confirming that the correlated-response mechanism operates regardless of which trait is under direct selection.

Under tight selection ($V_s = 0.005$), selecting on the high-V_M trait constrains drift of the nonselected partner to ≈ 13% of the neutral rate, whereas selecting on the low-V_M trait constrains drift of the nonselected partner to only ≈ 51% of the neutral rate—a roughly four-fold difference in effective constraint (Table S1). Under weak selection ($V_s = 0.1$), however, the two scenarios produce similar drift ratios (≈ 0.62–0.64), indicating that the asymmetry diminishes when selection is not strongly constraining.

The alignment between M and selection is quantitatively favorable but not qualitatively necessary. Selection is more efficient at constraining correlated traits when it targets the direction of greatest mutational input. In the empirical *Drosophila* system, the observation that wing size carries the largest mutational variance and is the likely target of selection is therefore favorable for the single-axis selection model, but the mechanism would still operate—albeit with weaker constraint—if stabilizing selection targeted a trait with lower mutational variance.

Table S1. Drift rate of the nonselected trait relative to neutral. The drift rate ratio is the rate of increase in among-replicate variance of the nonselected trait mean, divided by the corresponding rate under the fully neutral baseline. Values less than 1 indicate that selection constrains drift of the correlated trait. Under strong selection ($V_s = 0.005$), the constraint is roughly four-fold stronger when the high-V_M trait is selected; under weak selection ($V_s = 0.1$), both scenarios yield similar drift ratios.

Scenario	V_s	Drift ratio
Select high-V_M trait	0.005	0.13
Select high-V_M trait	0.1	0.64
Select low-V_M trait	0.005	0.51
Select low-V_M trait	0.1	0.62

Appendix B: Expected G under mutation–selection balance

This appendix reviews the classical theory for predicting the equilibrium genetic variance-covariance matrix $\mathbf{G}$ under mutation-selection balance. Importantly, these results assume that stabilizing selection acts on all traits, yielding an invertible selection matrix $\mathbf{S}$. Under the single-axis selection model, where selection targets only a single primary trait while others are neutral, $\mathbf{S}$ is rank-deficient and these analytical solutions do not directly apply. Nevertheless, understanding the traditional framework clarifies why alternative approaches (such as simulation) are necessary for the apparent selection regime.

B.1 Traditional Framework: Equilibrium in Infinite Populations. The structure of the additive genetic variance-covariance matrix ($\mathbf{G}$) is determined by the interplay between mutational input ($\mathbf{M}$), the selection matrix ($\mathbf{S}$), and effective population size (N_e). $\mathbf{M}$ is the mutational variance-covariance matrix describing per-generation mutational input; $\mathbf{S}$ is the selection matrix ($\mathbf{S} = \Omega^{-1}$ where Ω describes the width of the fitness peak; univariate $\Omega = V_s$). In an infinite population, $\mathbf{G}$ reaches a deterministic equilibrium where the input of variance equals the erosion by selection. Assuming multivariate stabilizing selection:

$$W(z) = \exp\left[-\frac{1}{2}(z-\theta)^T \mathbf{S}(z-\theta)\right] \quad [24]$$

Depending on the distribution of mutational effects, there are two primary regimes when deriving the equilibrium $\mathbf{G}$:

1. The Gaussian Regime: Under a regime of common mutations of weak effect (1, 10–13), the equilibrium $\mathbf{G}$ is found by solving:

$$\mathbf{M} - \mathbf{GSG} = \mathbf{0} \tag{25}$$

In this regime, the structure of $\mathbf{G}$ is a compromise between the bias of mutation ($\mathbf{M}$) and the demands of the fitness landscape ($\mathbf{S}$). The exact solution is given by the matrix geometric mean (12):

$$\mathbf{G} \approx \mathbf{S}^{-1/2} \left(\mathbf{S}^{1/2}\mathbf{M}\mathbf{S}^{1/2}\right)^{1/2} \mathbf{S}^{-1/2} \tag{26}$$

2. The House-of-Cards Regime: In the limit of rare mutations of large effect (1, 10, 13–15), the expected $\mathbf{G}$ for the House-of-Cards regime in an infinite population is proportional to the expectation of $\frac{\alpha\alpha^T}{\alpha^T S \alpha}$ over the distribution of mutational effects α (16):

$$\mathbf{G} \propto \mathbb{E}[\frac{\alpha\alpha^T}{\alpha^T \mathbf{S} \alpha}] \tag{27}$$

B.2 Traditional Framework: Finite Population with Drift. In finite populations ($N_e < \infty$), the production of genetic variance due to mutational input is balanced by both selection and genetic drift, which leads to a mutation-selection-drift balance. Under the Gaussian regime with non-overlapping generations, for a single haploid locus, this gives:

$$\mu\mathbf{M} \ = \ \bar{\mathbf{G}}\mathbf{S}\bar{\mathbf{G}} \ + \ \frac{1}{2N_e}\,\bar{\mathbf{G}} \tag{28}$$

where $\bar{\mathbf{G}}$ is the (expected) genetic covariance matrix over the stochastic evolutionary processes due to genetic drift.

Ref. (17) derived the expected genetic covariance matrix:

$$\bar{\mathbf{G}} \ = \ \mathbf{S}^{-1/2} \left[-\frac{1}{4N_e}\mathbf{I} + \frac{1}{2}\left(\frac{1}{(2N_e)^2}\mathbf{I}^2 + 4\mathbf{S}^{1/2}\mu\mathbf{M}\mathbf{S}^{1/2} \right)^{1/2} \right] \mathbf{S}^{-1/2}. \tag{29}$$

Limitation for the single-axis selection model: All of the above solutions require $\mathbf{S}^{-1}$ to exist. Under the single-axis selection model, selection acts only on a single primary trait (e.g., wing size), while all other traits are effectively neutral. This yields a rank-deficient selection matrix of the form:

$$\mathbf{S} = \begin{pmatrix} s_{11} & 0 \\ 0 & \mathbf{0} \end{pmatrix},$$

where the $(n-1) \times (n-1)$ block of zeros reflects the absence of direct selection on the remaining traits. Because $\mathbf{S}$ is singular, the analytical solutions above cannot be applied directly. Instead, I rely on individual-based simulations to characterize equilibrium $\mathbf{G}$ under the single-axis selection regime (see main text).

B.3 Interpreting empirical measurements of G and M. The observations from ref. (18) indicate that the off-diagonal elements in M are stronger than in G. The difference in magnitude between genetic and mutational trait covariances was reflected in differences in the relative magnitudes of the eigenvalues of the respective matrices: M has uneven eigenvalues (high eccentricity) compared to G, which exhibits relatively equal eigenvalues (low eccentricity). Let λ_M and λ_G be the eigenvalues of the respective matrices:

$$\frac{\lambda_{M,1}}{\lambda_{M,5}} > \frac{\lambda_{G,1}}{\lambda_{G,5}} \tag{30}$$

Reanalyzing G and M from ref. (19) shows similar patterns of eigenvalue distribution, where the eigenvalues of M are uneven compared to G (Fig. S4). Given that inbreeding might alter G (20), it should be noted that G was obtained from an outbred *D. melanogaster* population whereas M was estimated in inbred genotypes.

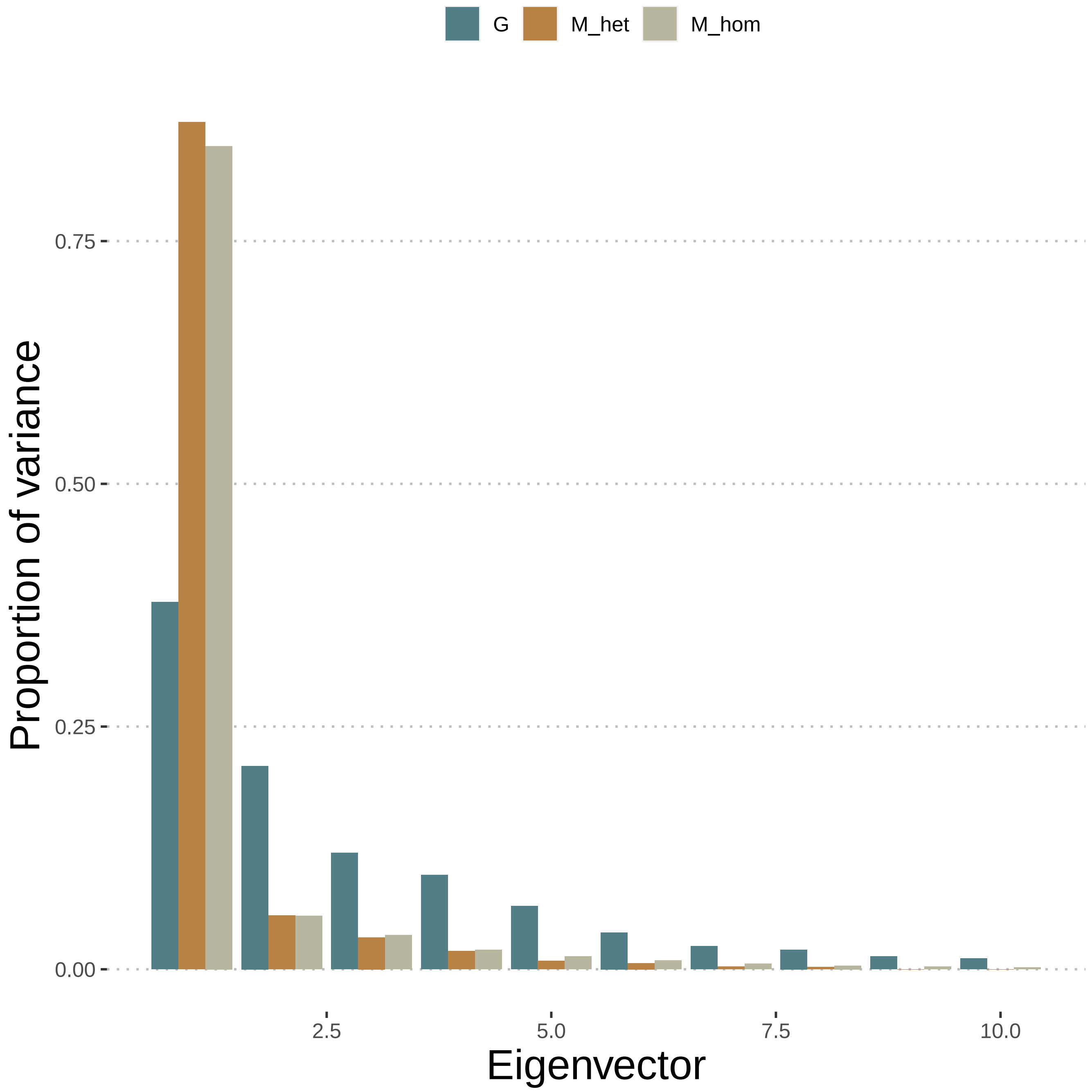

Fig. S4. Mutational variance is more eccentric than genetic variance in *Drosophila* wings. Eigenvalue distribution for G, M_{hom} (homozygous), and M_{het} (heterozygous) from ref. (19).

Appendix C: Robustness of alignment among G, R, and M

C.1: Sensitivity to selection strength and reference matrix. The alignment results are robust across a range of selection intensities and reference matrices. Below, I show that common subspace alignment is retained under weak stabilizing selection on wing size (Fig. S5) and when the empirical G matrix is used as the reference matrix instead of M (Fig. S6).

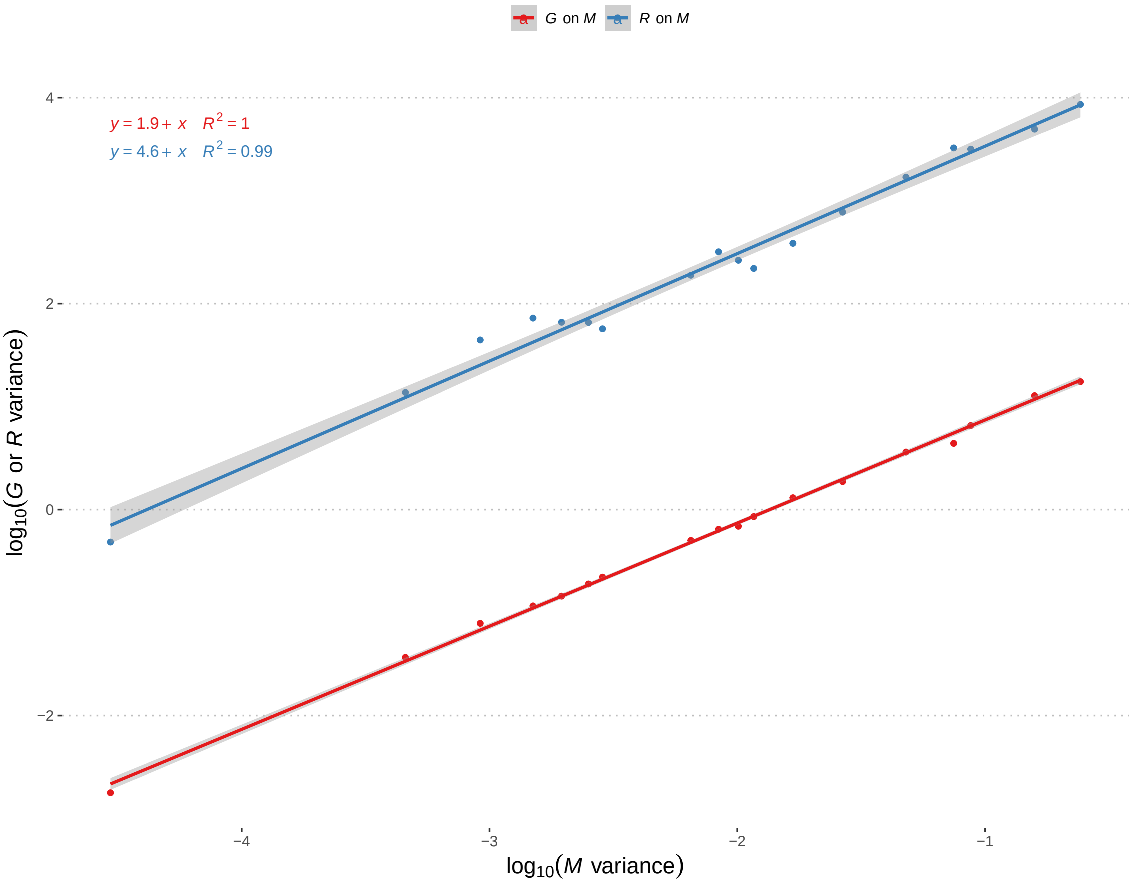

Fig. S5. Matrix alignment under weak stabilizing selection on wing size ($V_s = 0.5$). Common subspace analysis comparing R (blue) and G (red) to M. Points represent $\log_{10}$ variance along eigenvectors of M; lines show OLS regression. Wing size excluded. Shaded areas: 95% CI.

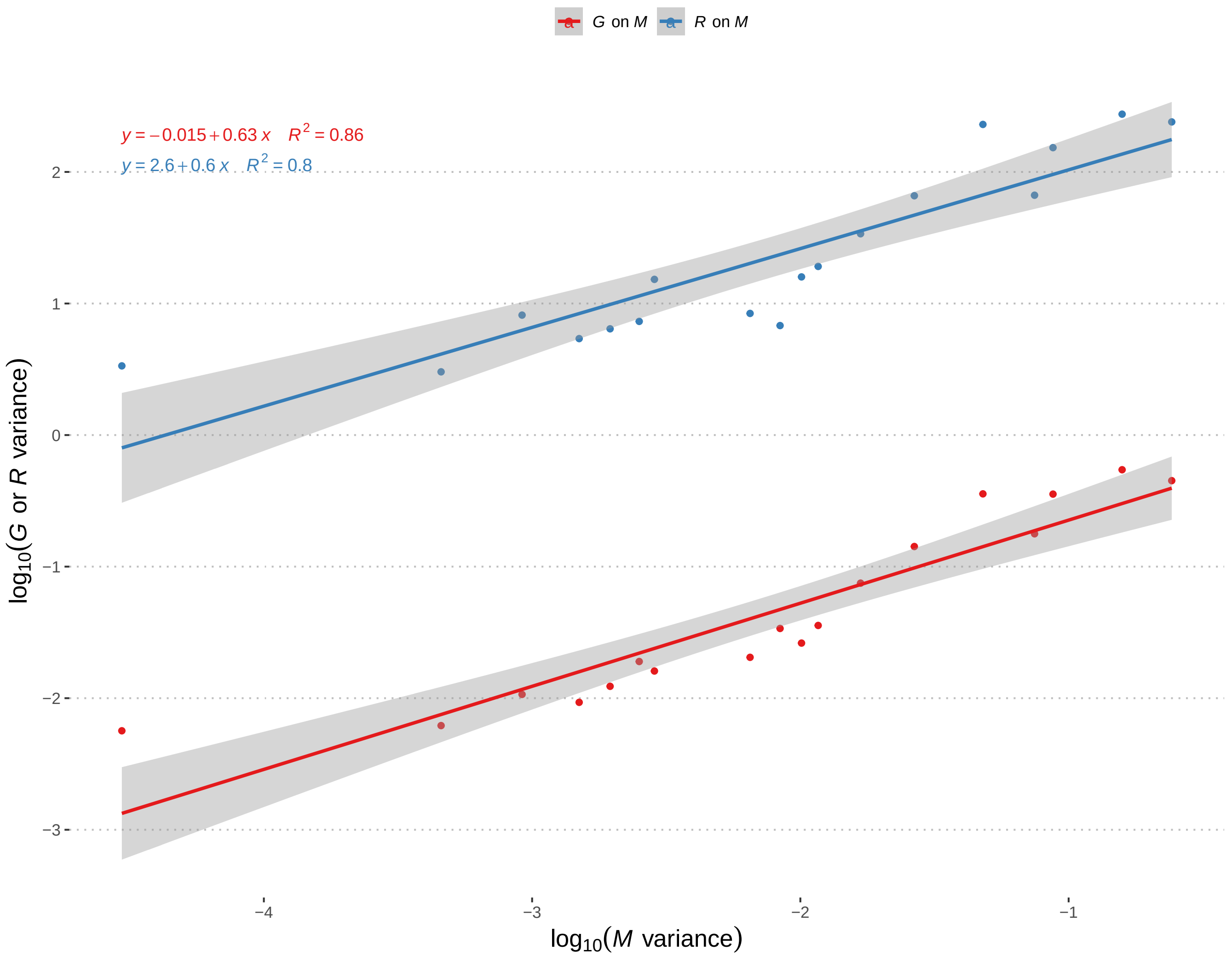

Fig. S6. Matrix alignment using the empirical G as the reference matrix. Common subspace analysis comparing the simulated divergence matrix R (blue) and simulated genetic variance matrix G (red) to the mutational variance matrix M, using the empirical G matrix from ref. (19) to replace M as the reference matrix. Points represent $\log_{10}$ variance of R, G, and M projected along the eigenvectors of the empirical G; lines show OLS regression. Wing size excluded. Shaded areas: 95% CI.

C.2: Selection on a low-variance shape trait: full 25-trait test. I repeated the full 25-trait simulation using the complete empirical M matrix from ref. (19) but with stabilizing selection targeting shape trait 1 instead of wing size. All other simulation parameters remained identical ($N = 500$, $V_s = 0.05$, $\sigma = 0.0001$, 20 replicate populations).

Results. Two key patterns emerge from this analysis: First, the alignment among G, M, and R is fully retained when selection targets shape trait 1 (Fig. S7), exactly as it is when selection targets wing size. This demonstrates the apparent selection mechanism is general. Second, the lowest G/M ratio shifts to trait 1 (Fig. S8), confirming that this depletion profile is a reliable diagnostic for identifying the specific target of selection.

Together with the two-trait analysis in Section A.5, these results confirm the generality of apparent selection while highlighting the G/M profile as a key differentiating test. The empirical finding that wing size exhibits the lowest G/M ratio directly supports selection on size and contradicts selection on shape trait 1 (or any other individual shape trait by extension).

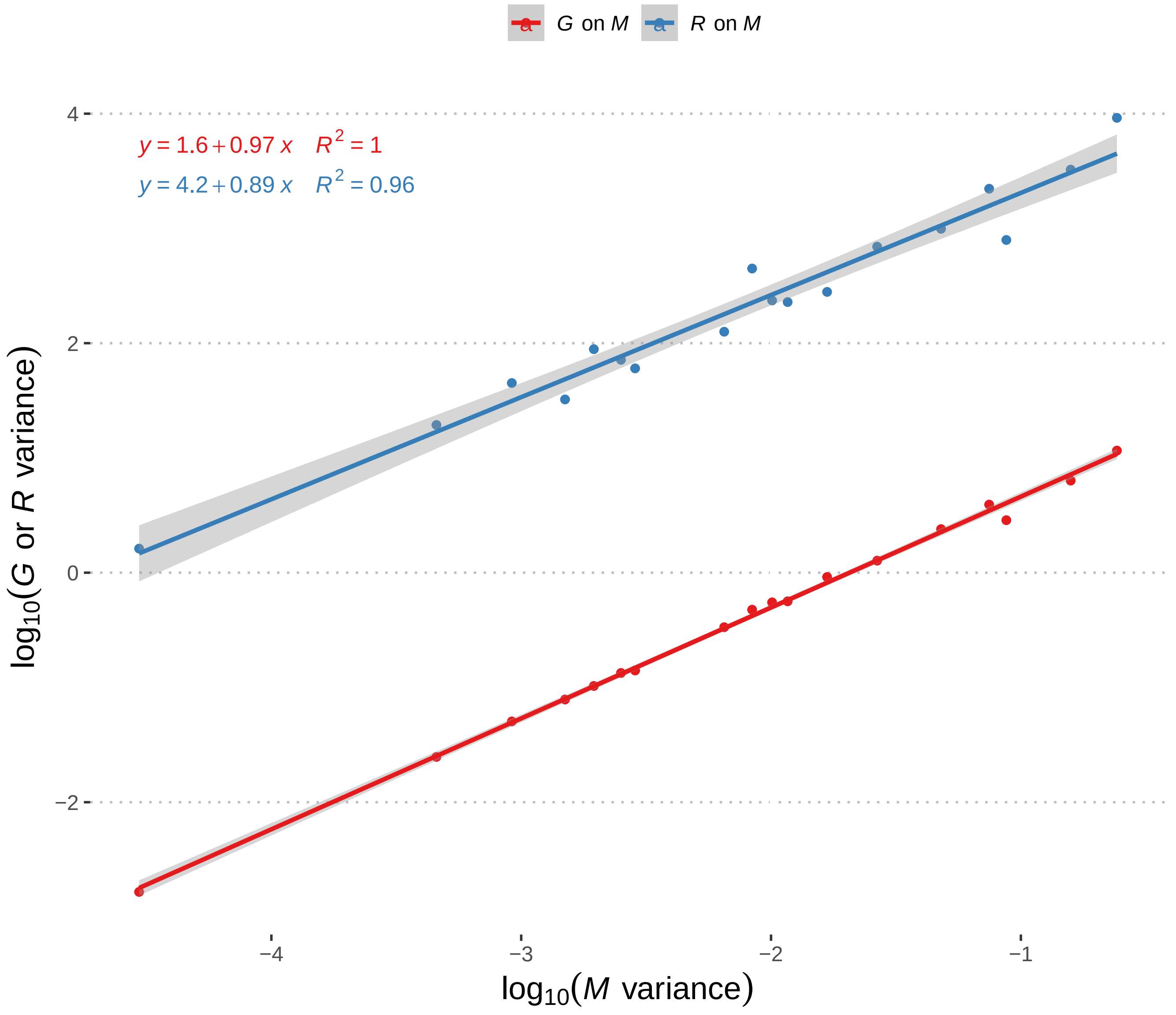

Fig. S7. Matrix alignment is retained when selection targets a low-variance shape trait. Common subspace analysis comparing R (blue) and G (red) to M when stabilizing selection targets shape trait 1 (lowest mutational variance) instead of wing size. Points represent $\log_{10}$ variance along eigenvectors of M; lines show OLS regression. Wing size included as a nonselected trait. Shaded areas: 95% CI. The strong positive alignment between R, G, and M is fully preserved, demonstrating that the apparent selection mechanism is general and does not depend on which trait is under direct selection. Simulation parameters: $N = 500$, $V_s = 0.05$, $\sigma = 0.0001$, 20 replicate populations.

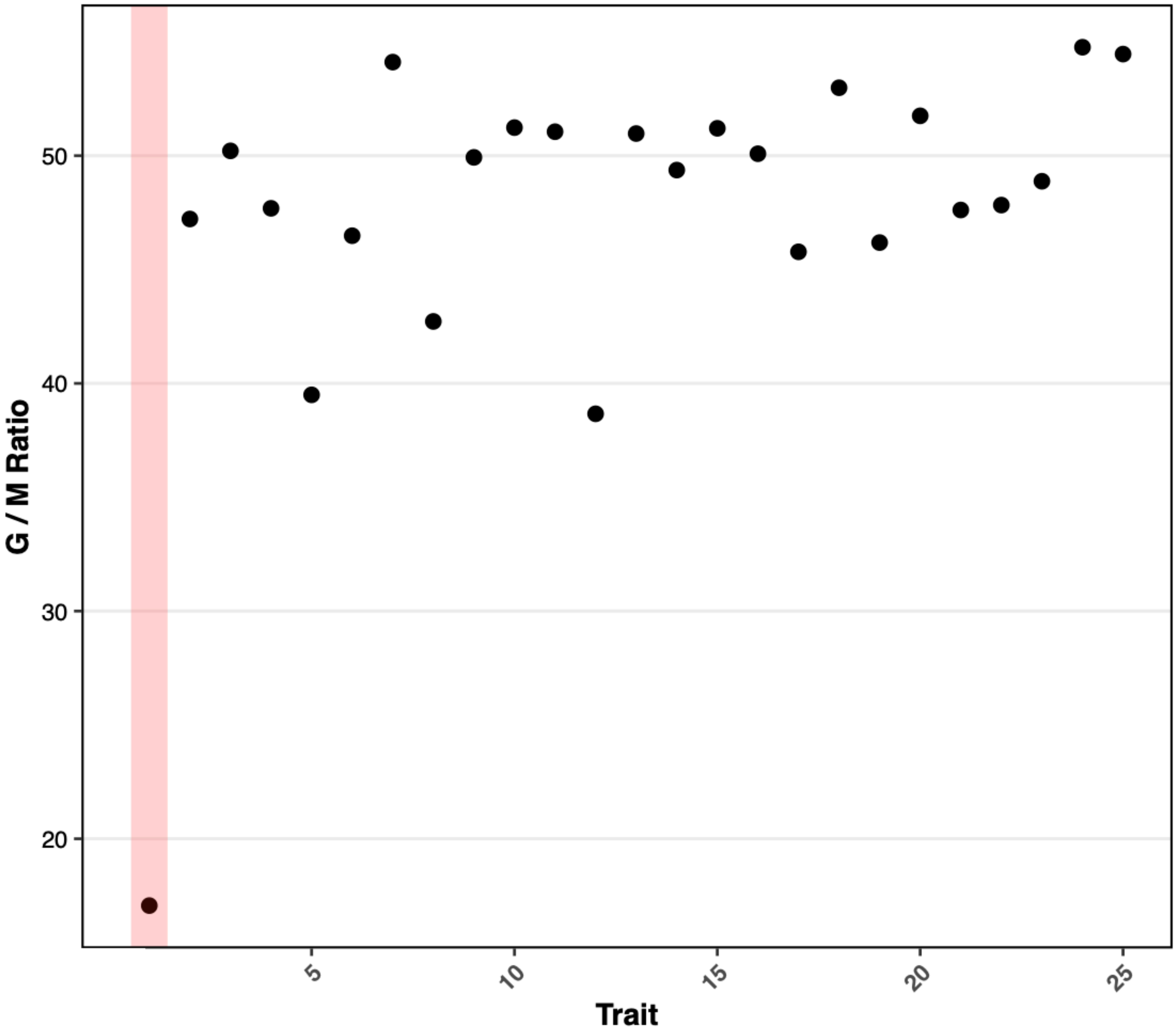

Fig. S8. G/M **ratio profile shifts to the selected trait under selection on shape trait 1.** Ratio of genetic to mutational variance (G/M) across all 25 traits when stabilizing selection targets shape trait 1 instead of wing size. The focal selected trait (trait 1) is highlighted, showing the lowest G/M ratio.

Appendix D: Comparison of G Between *Sepsis* and *Drosophila*

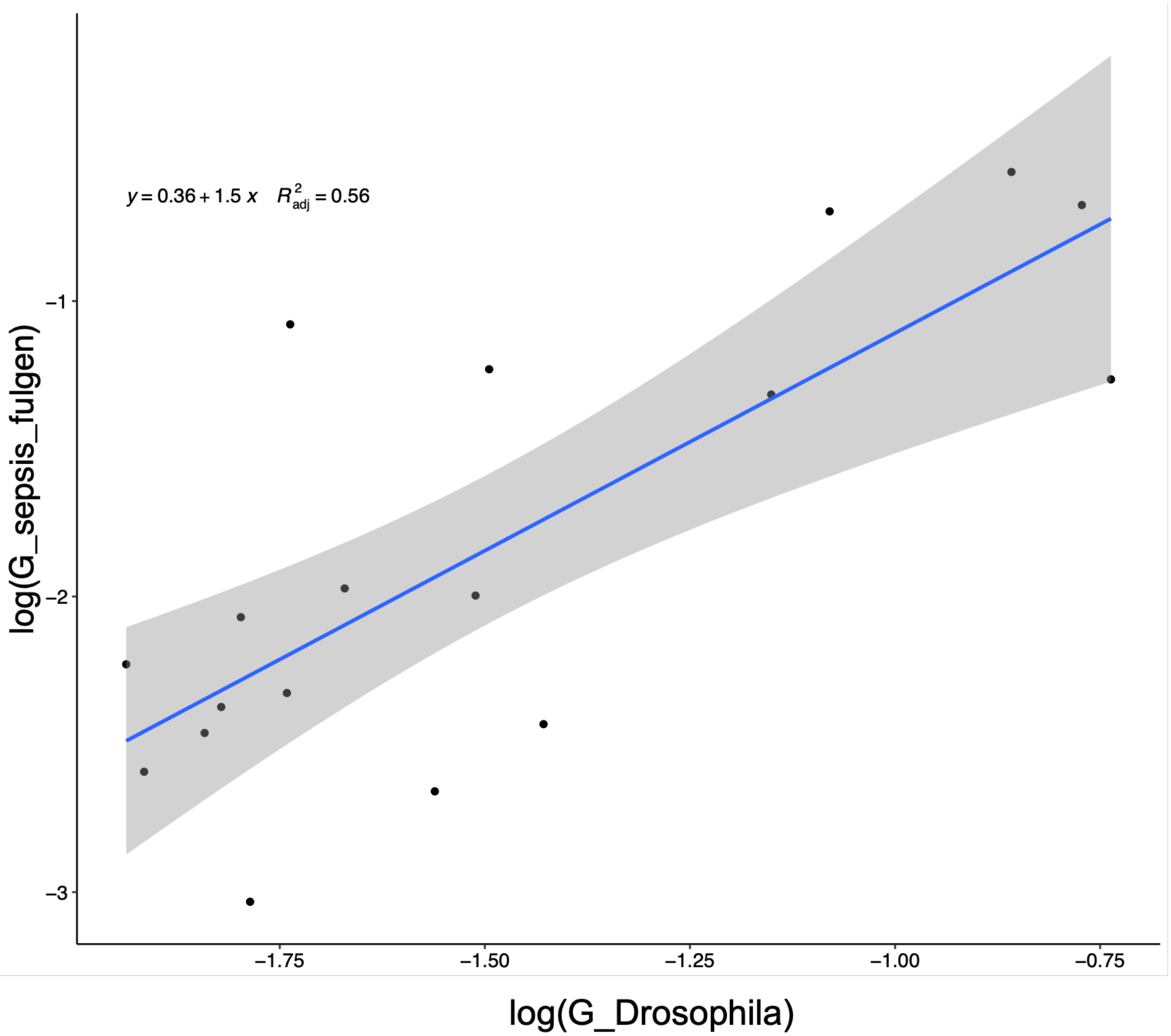

Fig. S9. Conservation of G structure between *Drosophila* and *Sepsis fulgens* (64 Mya divergence). Common subspace analysis using G of *Sepsis punctum* as the reference matrix. Data from ref. (21).

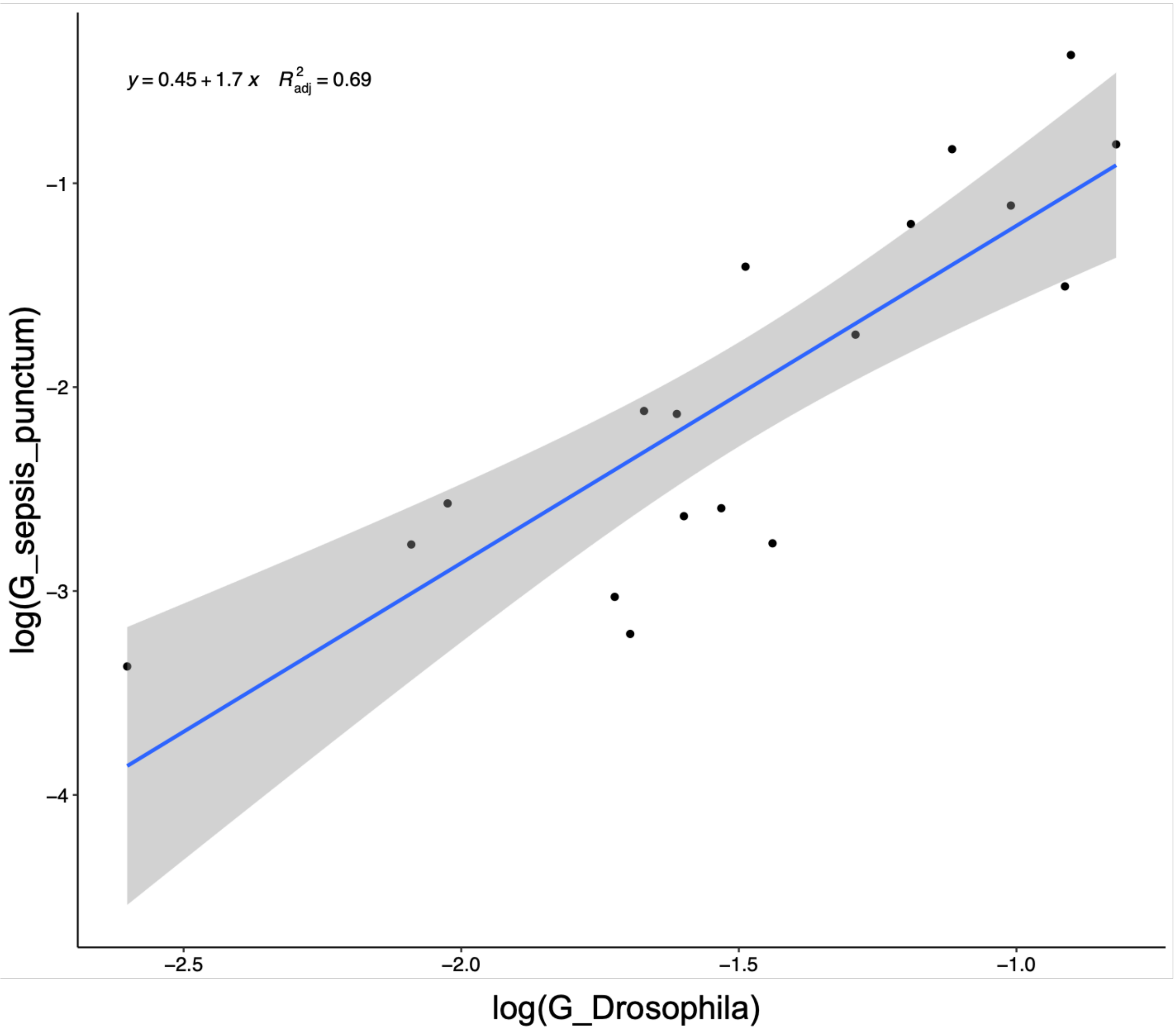

Fig. S10. Conservation of G structure between *Drosophila* and *Sepsis punctum* (64 Mya divergence). Common subspace analysis using G of *Sepsis fulgens* as the reference matrix. Data from ref. (21).

Appendix E: Polygenic basis of fly wing shape and vein positioning

Table S2. Summary of studies on the genetic architecture of fly wings

Study	Species / Sample	Method / Design	Key Findings
(22)	D. melanogaster — DGRP	Multivariate GWAS	Thousands of SNPs (many small effects)
(23)	D. melanogaster — RILs	QTL mapping	~Dozens of QTL
(24)	D. melanogaster — 191 P-element lines	Candidate-gene screen	Many candidate genes with small effects
(25)	D. melanogaster — natural variation panels	QTL / association	Several QTL/loci detected
(26)	Review / synthesis	Literature Review	Polygenic & context-dependent (synthesis)
(27)	D. melanogaster — functional replication	Artificial selection	Replicated multivariate shape changes
(28)	D. melanogaster — seasonal sampling	Population sampling	Seasonal shape variation detected
(29)	D. melanogaster — developmental experiments	Developmental genetics	Mechanistic link: growth & morphogens influence shape

Abbreviations: DGRP: Drosophila Genetic Reference Panel, a collection of fully sequenced inbred lines derived from a natural population; RIL: Recombinant Inbred Lines, homozygous lines derived from crossing two parental strains followed by repeated selfing or sib-mating; QTL: Quantitative Trait Locus, a genomic region associated with variation in a quantitative trait; SNP: Single Nucleotide Polymorphism, a single base-pair variant in the genome; GWAS: Genome-Wide Association Study, a method to identify genetic variants associated with trait variation by scanning across the entire genome.

Appendix F: Eccentricity analysis — supplementary figures

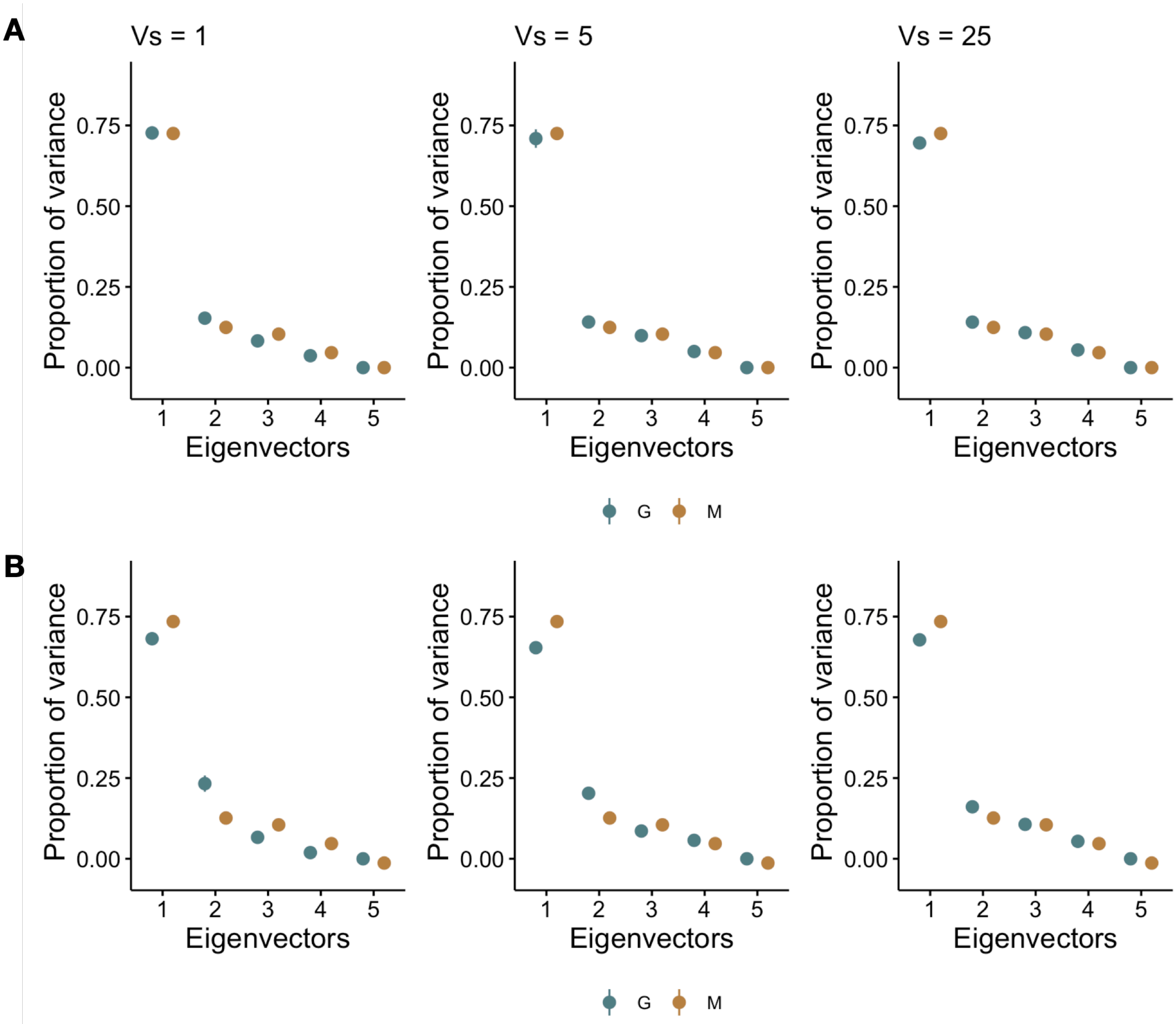

Fig. S11. Eigenvalue spectra of equilibrium G versus M under single-axis selection and correlational selection. Each panel compares the proportion of total variance explained by the first five eigenvectors for the simulated equilibrium G (teal) and the empirical M matrix (gold) from ref. (18). Columns correspond to increasing stabilizing selection width ($V_s = 1$, 5, and 25). (A) Single-axis selection: stabilizing selection acts only on wing size. Under strong selection ($V_s = 1$), G becomes visibly less eccentric than M—the leading eigenvalue is reduced and variance is redistributed among trailing eigenvectors. As selection weakens ($V_s = 25$), G converges toward M. (B) Correlational selection: the selection matrix S is proportional to M^{-1}. Here, G retains eigenvalue proportions close to those of M across all selection strengths, because multivariate stabilizing selection erodes variance proportionally in all directions. Error bars show ± 1 s.d. across 5 replicates ($N = 500$).

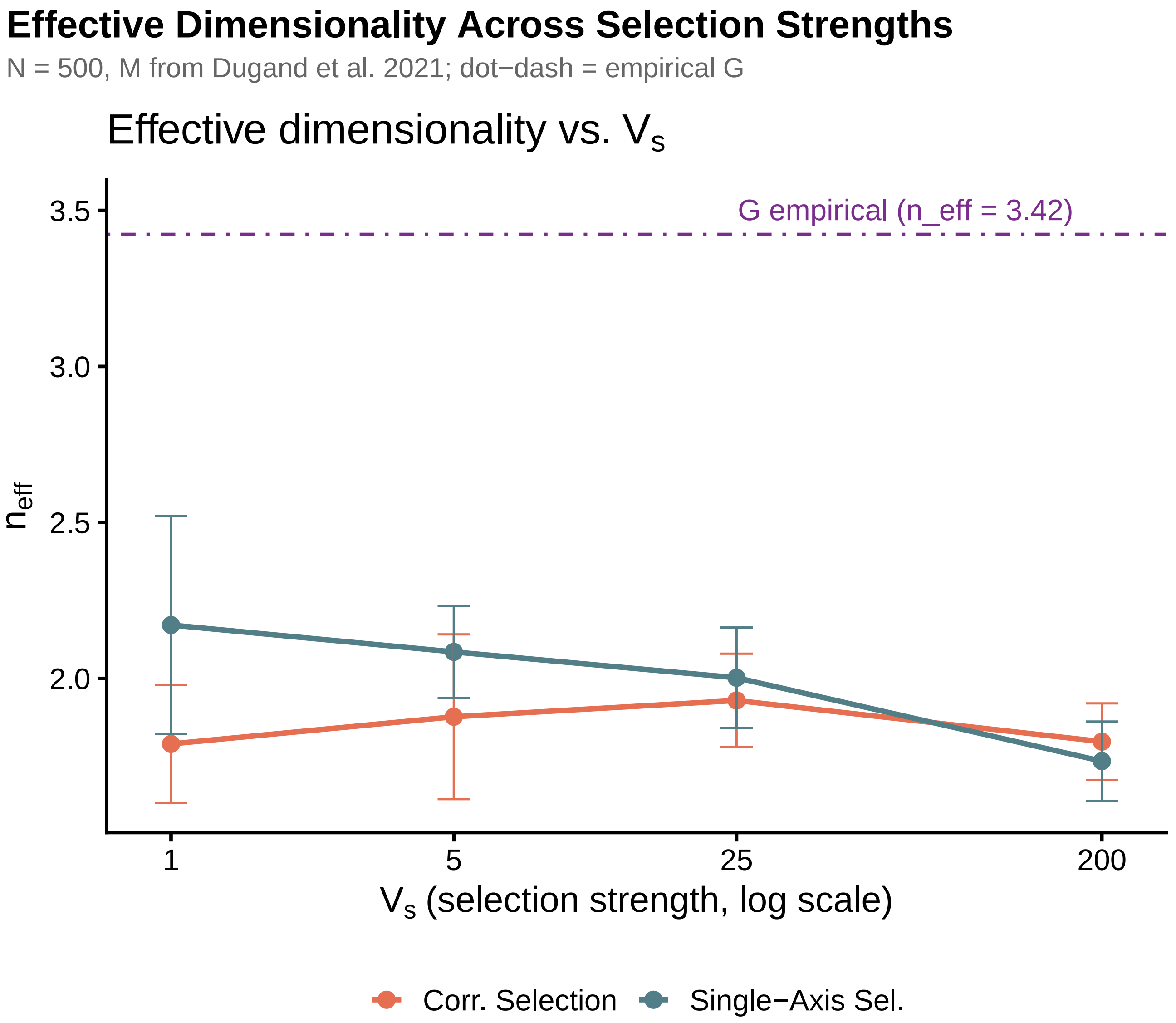

Fig. S12. Effective dimensionality across selection strengths. Effective dimensionality (n_{eff}) as a function of the stabilizing selection width V_s (log scale). Lines show mean $\pm$ s.d. across 5 replicates for single-axis selection (teal) and correlational selection (orange). Dashed brown line: M baseline; dot-dash purple line: empirical G from ref. (18). The empirical $n_{\text{eff}} = 3.42$ exceeds all simulated single-axis selection values, indicating additional sources of genetic dimensionality.

Supplementary References

References

1. T Johnson, N Barton, Theoretical models of selection and mutation on quantitative traits. *Philos. Transactions Royal Soc. B: Biol. Sci.* **360**, 1411–1425 (2005).
2. A Robertson, R Brink, Heritage from mendel. *Madison, Wis.* pp. 265–280 (1967).
3. B Walsh, M Lynch, *Evolution and selection of quantitative traits.* (Oxford University Press), (2018).
4. K McGuigan, L Rowe, MW Blows, Pleiotropy, apparent stabilizing selection and uncovering fitness optima. *Trends Ecol. & Evol.* **26**, 22–29 (2011).
5. PD Keightley, WG Hill, Variation maintained in quantitative traits with mutation–selection balance: pleiotropic side-effects on fitness traits. *Proc. Royal Soc. London. Ser. B: Biol. Sci.* **242**, 95–100 (1990).
6. NH Barton, Pleiotropic models of quantitative variation. *Genetics* **124**, 773–782 (1990).
7. A Singh, AF Agrawal, Sex-specific variance in fitness and the efficacy of selection. *The Am. Nat.* **199**, 587–602 (2022).
8. JE Taylor, The genealogical consequences of fecundity variance polymorphism. *Genetics* **182**, 813–837 (2009).
9. R Bürger, M Lynch, Evolution and extinction in a changing environment: a quantitative-genetic analysis. *Evolution* **49**, 151–163 (1995).

10. R Bürger, *The mathematical theory of selection, recombination, and mutation.* (John Wiley & Sons), (2000).
11. M Kimura, A stochastic model concerning the maintenance of genetic variability in quantitative characters. *Proc. Natl. Acad. Sci.* **54**, 731–736 (1965).
12. R Lande, The genetic covariance between characters maintained by pleiotropic mutations. *Genetics* **94**, 203–215 (1980).
13. M Bulmer, Maintenance of genetic variability by mutation–selection balance: a child's guide through the jungle. *Genome* **31**, 761–767 (1989).
14. M Turelli, Heritable genetic variation via mutation-selection balance: Lerch's zeta meets the abdominal bristle. *Theor. population biology* **25**, 138–193 (1984).
15. M Turelli, Effects of pleiotropy on predictions concerning mutation-selection balance for polygenic traits. *Genetics* **111**, 165–195 (1985).
16. XS Zhang, WG Hill, Multivariate stabilizing selection and pleiotropy in the maintenance of quantitative genetic variation. *Evolution* **57**, 1761–1775 (2003).
17. S Chantepie, LM Chevin, How does the strength of selection influence genetic correlations? *Evol. letters* **4**, 468–478 (2020).
18. RJ Dugand, JD Aguirre, E Hine, MW Blows, K McGuigan, The contribution of mutation and selection to multivariate quantitative genetic variance in an outbred population of drosophila serrata. *Proc. Natl. Acad. Sci.* **118**, e2026217118 (2021).
19. D Houle, GH Bolstad, K van der Linde, TF Hansen, Mutation predicts 40 million years of fly wing evolution. *Nature* **548**, 447–450 (2017).
20. PC Phillips, MC Whitlock, K Fowler, Inbreeding changes the shape of the genetic covariance matrix in drosophila melanogaster. *Genetics* **158**, 1137–1145 (2001).
21. PT Rohner, D Berger, Macroevolution along developmental lines of least resistance in fly wings. *Nat. Ecol. & Evol.*. 9, 639–651 (2025).
22. W Pitchers, et al., A multivariate genome-wide association study of wing shape in drosophila melanogaster. *Genetics* **211**, 1429–1447 (2019).
23. E Zimmerman, A Palsson, G Gibson, Quantitative trait loci affecting components of wing shape in drosophila melanogaster. *Genetics* **155**, 671–683 (2000).
24. VP Carreira, IM Soto, J Mensch, JJ Fanara, Genetic basis of wing morphogenesis in drosophila: sexual dimorphism and non-allometric effects of shape variation. *BMC developmental biology* **11**, 32 (2011).
25. JG Mezey, D Houle, SV Nuzhdin, Naturally segregating quantitative trait loci affecting wing shape of drosophila melanogaster. *Genetics* **169**, 2101–2113 (2005).
26. A Matamoro-Vidal, I Salazar-Ciudad, D Houle, Making quantitative morphological variation from basic developmental processes: Where are we? the case of the drosophila wing. *Dev. Dyn.* **244**, 1058–1073 (2015).
27. K Pelletier, Quantitative variation in drosophila melanogaster wing shape and size (2023).
28. BŞ Önder, CF Aksoy, Seasonal variation in wing size and shape of drosophila melanogaster reveals rapid adaptation to environmental changes. *Sci. Reports* **12**, 14622 (2022).
29. J Parker, G Struhl, Control of drosophila wing size by morphogen range and hormonal gating. *Proc. Natl. Acad. Sci.* **117**, 31935–31944 (2020).
